# Neonatal brain structure, cognitively stimulating parenting and behavioural outcomes in preschool children with congenital heart disease and controls

**DOI:** 10.1038/s41598-026-55283-5

**Published:** 2026-05-29

**Authors:** Barat Gal-Er, Alexandra F. Bonthrone, Andrew T.M. Chew, Sian Wilson, Daniel Cromb, Alexia Egloff, Kuberan Pushparajah, John Simpson, Mirthe E.M. van der Meijden, Mary A. Rutherford, Joseph V. Hajnal, A. David Edwards, Jonathan O’Muircheartaigh, Chiara Nosarti, Serena J. Counsell

**Affiliations:** 1 Centre for the Developing Brain, Research Department of Early Life Imaging, School of Biomedical Engineering and Imaging Sciences, King’s College London, SE1 7EH London, UK; 2 Department of Computer Science, University of Oxford, Oxford, UK; 3 Department of Cardiovascular Imaging, School of Biomedical Engineering and Imaging Sciences, King’s College London, London, UK; 4 Department of Fetal and Paediatric Cardiology, Evelina London Children’s Hospital, London, UK; 5 Research Department of Imaging Physics & Engineering, School of Biomedical Engineering and Imaging Sciences, King’s College London, London, UK; 6 Department for Forensic and Neurodevelopmental Sciences, Institute of Psychiatry, Psychology and Neuroscience, King’s College London, London, UK; 7 MRC Centre for Neurodevelopmental Disorders, King’s College London, London, UK; 8 Department of Child and Adolescent Psychiatry, Institute of Psychiatry, Psychology and Neuroscience, King’s College London, London, UK

**Keywords:** congenital heart disease, neonatal, brain, MRI, structural covariance, neurodevelopmental outcomes, Diseases, Medical research, Neuroscience

## Abstract

Children with congenital heart disease (CHD) are at increased risk of altered early brain development and neurodevelopmental impairments. Although environmental factors are known to influence neurodevelopmental outcomes, the interplay between neonatal brain structure and the home environment in shaping behavioural outcomes remains unclear. We investigated associations between neonatal structural covariance networks (SCNs), cognitively stimulating parenting, and behavioural outcomes at 4–6 years in 44 preschool children with CHD and 117 controls. Principal component analysis of 19 parent-reported questionnaires identified distinct components of childhood behaviour. Parents completed the cognitively stimulating parenting scale (CSPS) to assess cognitive stimulation at home. Forty SCNs were extracted from Jacobian determinants of neonatal T2-weighted MRI using independent component analysis. In children with CHD, anterior thalamus and cingulum morphometry was associated with empathy in childhood. Higher CSPS scores were associated with fewer neurodevelopmental difficulties in CHD, but not controls. In both groups, CSPS moderated the relationship between superior temporal gyrus morphometry and empathy while, in CHD only, CSPS moderated the relationship between inferior temporal gyrus morphometry and empathy. These findings identify early neurobiological and environmental determinants of behavioural outcomes in children with CHD and highlight the home environment as a modifiable factor in supporting neurodevelopment in this population.

**Supplementary Information:**

The online version contains supplementary material available at 10.1038/s41598-026-55283-5.

## Introduction

Congenital heart disease (CHD) is the most common congenital malformation, with a global prevalence of approximately 8 per 1,000 births^1^. Advances in surgical management and perioperative care have markedly improved survival, with approximately 90% of children now surviving into adulthood^2,3^. However, long-term neurodevelopmental sequelae have become increasingly apparent. Children with CHD frequently exhibit impairments across multiple domains from early childhood, including cognition, motor performance and language, alongside difficulties with executive functioning, social interaction, inattention and emotional problems that may persist into school age and beyond^4^. These deficits can range from mild to severe, and have a prolonged impact on quality of life^5^ and educational attainment^6^. In the United Kingdom, 50% of primary school children with CHD receive special educational needs provision^7^.

Magnetic resonance imaging (MRI) studies in CHD have demonstrated brain injury^8–11^ and altered early brain development, including smaller cortical grey matter^12–19^, deep grey matter^12,15–17^, white matter^12,16,17^ and cerebellar volumes^12,16,17,19,20^, reduced cortical folding^14,21,22^ and altered hippocampal subfield morphometry^23^ in the neonatal period. Such alterations in neonatal brain development have been linked to neurodevelopmental outcomes in both toddlerhood and in middle childhood, including in cognitive^15,17,24–26^ and behavioural^27^ domains. However, relatively few studies have characterised behavioural outcomes in children with CHD during the preschool years, a period of socio-emotional and behavioural development that underpins later learning, school readiness and academic success^28–30^. To our knowledge, the relationship between neonatal structural brain development and behavioural outcomes in preschool children with CHD remains largely unexplored.

One approach used to investigate neonatal structural brain development involves assessing the anatomical covariance of regional brain morphometry across individuals by delineating structural covariance networks (SCNs). This whole-brain, data-driven approach uses Jacobian determinants derived from T2-weighted MRI to reflect the relative expansion or contraction of brain regions, which can be decomposed using independent component analysis (ICA) into spatially distinct SCNs without the need for predefined regions of interest. This method has been applied to characterise structural brain maturation in typical development^31–33^, as well as alterations in clinical populations^34–36^. We have previously reported altered structural covariance compared to controls in fetuses^37^ and neonates with CHD before cardiac surgery^38^, involving deep grey matter structures, cingulate gyrus, corpus callosum, cerebellum and CSF spaces^38^.

The home environment and socioeconomic factors are known to shape cognitive and executive functioning in both healthy children^39,40^ and in children with CHD^41–44^. We have previously shown that in children with CHD a more cognitively stimulating home environment is associated with higher cognitive scores at 22 months^45^, better executive functioning^46^, and fewer ADHD symptoms and peer relationship problems^47^ in the preschool years. Additionally, in a large cohort of children born preterm, cognitively stimulating parenting was associated with decreased executive dysfunction at 4–7 years^36^. However, previous studies in the CHD population have focused on either the relationship between neonatal brain development and neurodevelopmental outcomes^15,17,48^, or on the relationship between the home environment and neurodevelopmental outcomes^45–47^. The role of the home environment in modifying the relationship between neonatal brain structure and neurodevelopmental outcomes in children with CHD has not been examined.

The aims of this study were to assess (i) whether there are associations between neonatal brain structure and behavioural outcomes at 4–6 years, and whether these associations differ between children with CHD and healthy controls, (ii) whether the relationship between a cognitively stimulating home environment and behavioural outcomes differs between children with CHD and controls, and (iii) whether the association between neonatal brain structure and behavioural outcomes is altered by the level of cognitive stimulation in the home in each group.

## Results

### Participant characteristics

44 participants with CHD and 117 control participants met the inclusion criteria and were included in the analyses. Infants with CHD were born at a younger gestational age (GA) (*p* < 0.001), scanned at a younger postmenstrual age (PMA) (*p* < 0.001), were younger at follow-up assessment (*p* = 0.001) and had lower maternal education (*p* = 0.002) compared to controls. The CHD group did not differ from the control group in other demographic variables (sex: *p* = 0.99; birth weight Z-score: *p* = 0.57; appropriate for gestational age: *p* = 0.924; index of multiple deprivation (IMD): *p* = 0.54) or in cognitively stimulating parenting scale (CSPS) scores (*p* = 0.06). Demographics for all participants are summarized in Table 1.

**Table 1:** Participant characteristics.

Gestational age at birth [weeks], median (IQR)	CHD (*N* = 44)	Controls (*N* = 117)	*p*-value
	38.71 (38.29–39.00)	40.43 (39.43–41.14)	< 0.001*
Postmenstrual age at scan [weeks], median (IQR)	39.29 (38.71–39.71)	42.43 (40.86–43.43)	< 0.001*
Male, N (%)	21 (48)	54 (46)	0.99
Birth weight Z-score, mean (SD)	-0.21 (0.96)	-0.11 (0.84)	0.568
Appropriate for gestational age, N (%)	37 (84)	101 (86)	0.924
Index of Multiple Deprivation Quintile, N (%)			
First (most deprived)	4 (9)	15 (13)	0.540
Second	8 (18)	33 (28)	
Third	10 (23)	24 (21)	
Fourth	13 (30)	23 (20)	
Fifth (least deprived)	9 (20)	22 (19)	
Maternal education, N (%)			
Below university degree	15 (34)	9 (8)	0.002*
University undergraduate	17 (39)	67 (57)	
University postgraduate	9 (20)	37 (32)	
Missing	3 (7)	4 (3)	
Primary cardiac defect, N (%)			
Abnormal streaming of blood			
TGA	21 (48)	-	-
TAPVD	1 (2)	-	-
Left-sided heart lesions			
CoA	9 (20)	-	-
HLHS	1 (2)	-	-
Aortic stenosis	2 (5)	-	-
Right-sided heart lesions			
ToF	4 (9)	-	-
Pulmonary stenosis	4 (9)	-	-
Pulmonary atresia	2 (5)	-	-
Age at assessment [months], median (IQR)	49.24 (48.45–50.65)	52.19 (48.70–62.55)	0.001*
CSPS, median (IQR)	37.0 (31.0–39.0)	38.0 (35.0–40.0)	0.06

*Significant result *p* < 0.05. Abbreviations: CoA, coarctation of the aorta; CSPS, cognitively stimulating parenting scale; HLHS, hypoplastic left heart syndrome; IQR, interquartile range; TAPVD, total anomalous pulmonary venous drainage; TGA, transposition of the great arteries; ToF, tetralogy of Fallot.

### Principal components of childhood behavioural outcomes

**Fig. 1:**
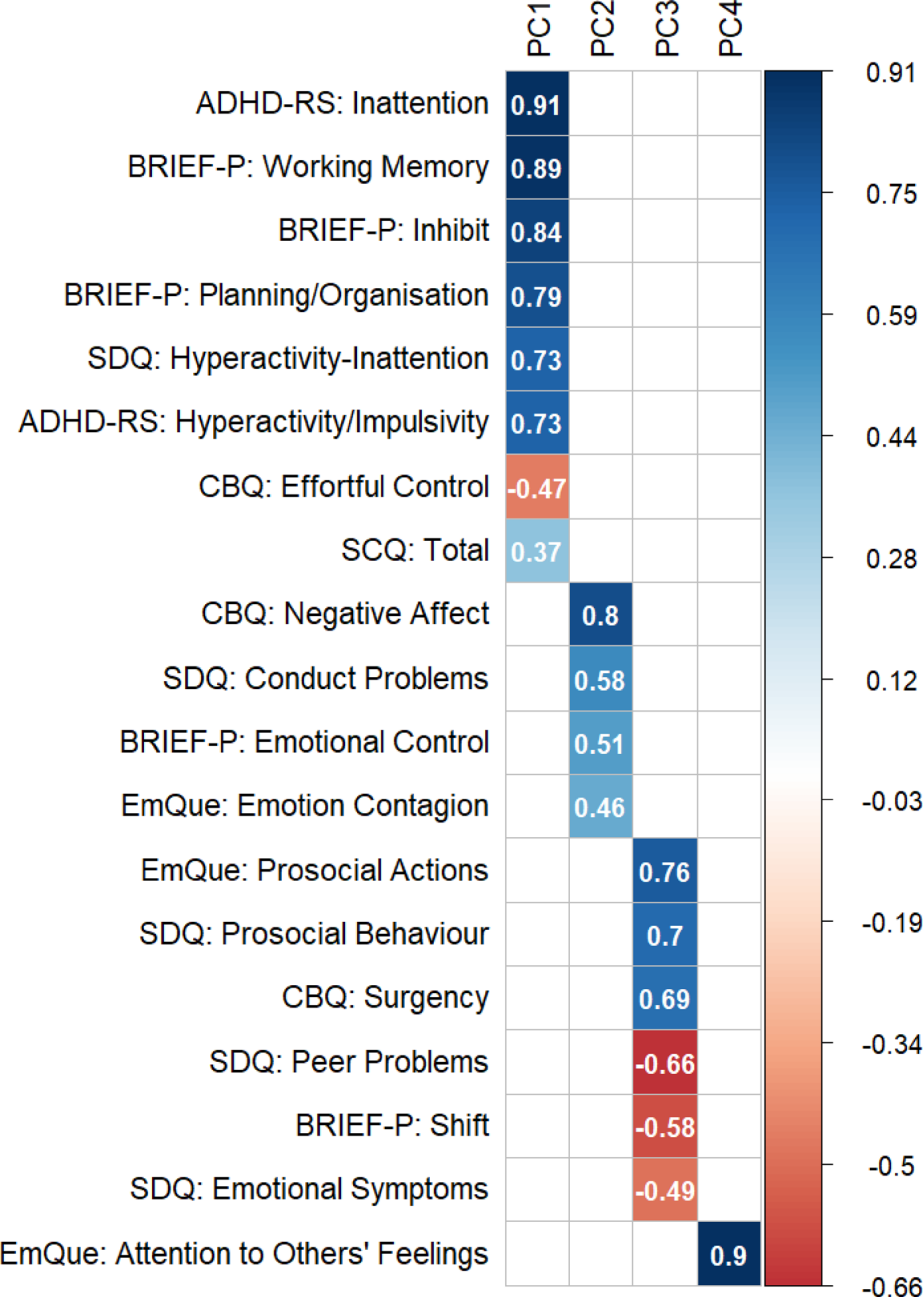
Heatmap of loadings of each questionnaire subscale on principal component (PC) 1, PC2, PC3, and PC4. Abbreviations: ADHD-RS, ADHD Rating Scale; BRIEF-P, Behaviour Rating Inventory of Executive Function, Preschool version; CBQ, Children’s Behaviour Questionnaire; EmQue, Empathy Questionnaire; SDQ, Strengths and Difficulties Questionnaire; SCQ, Social Communications Questionnaire.

The Kaiser-Meyer-Olkin Measure of Sampling Adequacy was 0.84 and the Bartlett’s Test of Sphericity was significant (χ^2^ = 1678.19, *p* < 0.001), showing that the sample size was adequate for a PCA^49^. The number of retained components was determined using Horn’s parallel analysis and visual inspection of the scree plot (Supplementary Fig. S1). Four components were retained and subjected to oblimin (oblique) rotation, given the expectation that components may be correlated. Together, these components explained a cumulative 64% of total variance and are depicted in Fig. 1.

PC1 was driven by positive loadings of questionnaire subscales capturing ADHD symptoms (Strengths and Difficulties Questionnaire [SDQ]: hyperactivity-inattention; ADHD Rating Scale [ADHD-RS]: inattention and hyperactivity), autism traits (Social Communications Questionnaire), and executive function deficits (Behaviour Rating Inventory of Executive Function, Preschool version [BRIEF-P]: inhibit, working memory, and planning/organizing), as well as negative loadings of a scale capturing self-regulation of behaviour and attention (Children’s Behaviour Questionnaire [CBQ]: effortful control). We termed this the “neurodevelopmental difficulties” component.

PC2 was driven by positive loadings of questionnaire subscales capturing negative affect (CBQ), difficulties regulating emotions (BRIEF-P: emotional control), emotion contagion (Empathy Questionnaire [EmQue]) and conduct problems (SDQ). We interpret this component as reflecting a measure of emotional and behavioural problems. We therefore termed it the “emotional dysregulation” component.

PC3 was driven by positive loadings of scales capturing a tendency towards positive affect and extraversion (CBQ: surgency) and prosocial behaviours (EmQue: prosocial actions; SDQ: prosocial behaviour), as well as negative loadings of a scale capturing difficulties with cognitive flexibility (BRIEF-P: shift), emotional problems (SDQ) and peer problems (SDQ). We interpret this component as reflecting outgoing, social and prosocial traits with fewer peer-related problems. We termed this the “social function” component.

PC4 was driven by positive loadings of a scale capturing empathetic behaviour (EmQue: attention to others’ feelings). We termed this the “empathy” component.

### Structural covariance networks (SCNs)

**Fig. 2:**
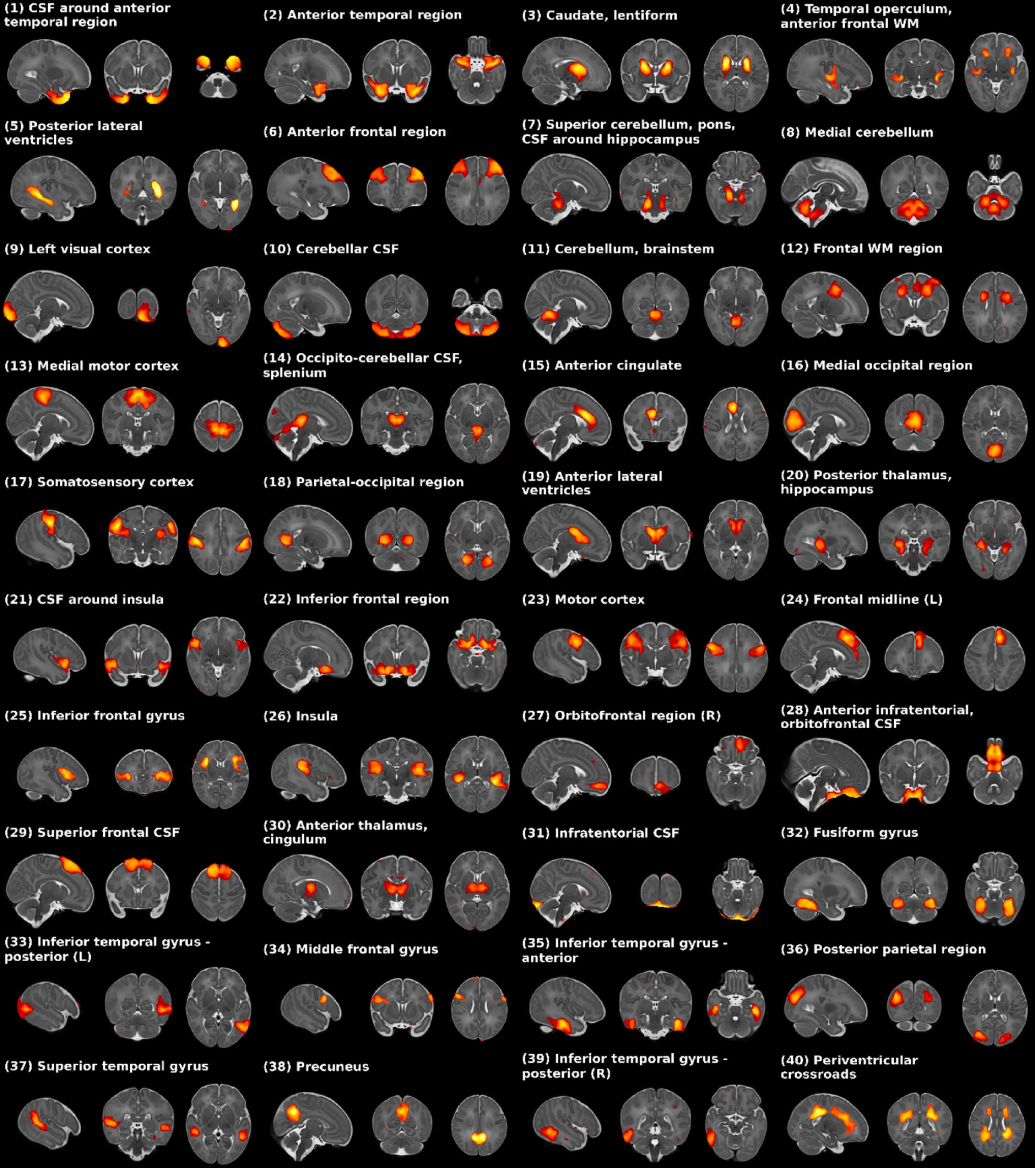
Neonatal structural covariance networks identified with independent component analysis (*n* = 40). Abbreviations: CSF, cerebrospinal fluid; L, left; R, right; WM, white matter.

Independent component analysis of neonatal Jacobian determinants was used to extract 40 SCNs, depicted in Fig. 2.

### Testing whether associations between neonatal SCNs and behavioural outcomes in childhood vary by group

**Fig. 3:**
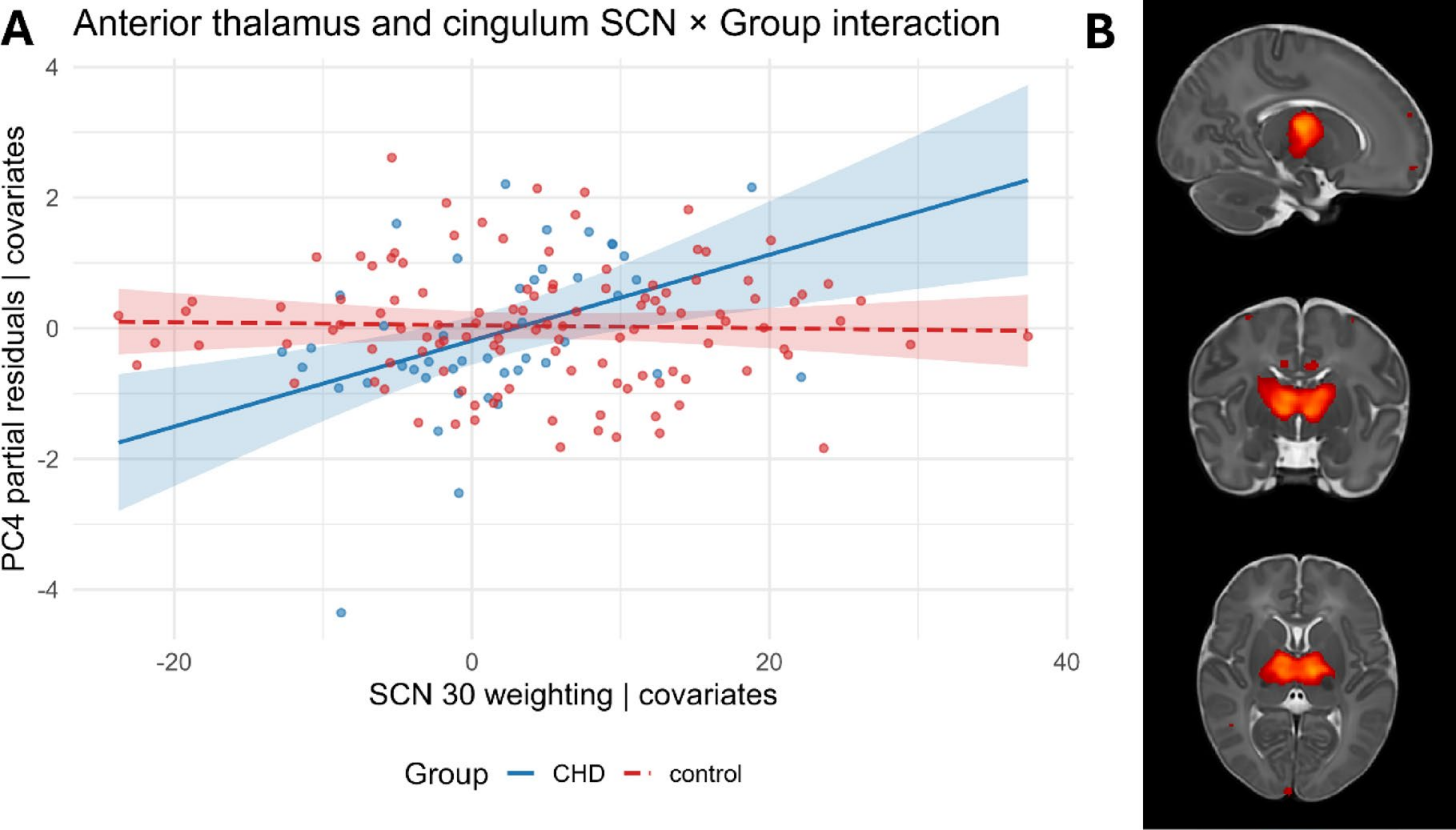
(**A**) Positive association between neonatal anterior thalamus and cingulum SCN (SCN 30) and empathy (PC4) in preschool children with CHD (blue) but not in controls (red), after adjusting for GA at birth, PMA at scan, sex, and IMD. (**B**) Visualization of SCN 30. Abbreviations: GA, gestational age; IMD, index of multiple deprivation; PC, principal component; PMA, postmenstrual age; SCN, structural covariance network.

The interaction between group and the SCN encompassing the anterior thalamus and cingulum (SCN 30) was significantly associated with empathy (PC4) in childhood, after adjusting for GA at birth, PMA at scan, sex and IMD (B [95% CI] -0.068 [-0.110 to -0.026], t = -3.202, pFDR = 0.008) (Fig. 3; Supplementary Table S1).

Post hoc analysis revealed that the SCN encompassing the anterior thalamus and cingulum (SCN 30) was significantly associated with empathy (PC4) in children with CHD (B [95% CI] 0.061 [0.012 to 0.011], t = 2.53, *p* = 0.015) but not in controls (-0.002 [-0.017 to 0.012], t = -0.30, *p* = 0.762).

No other significant group-SCN interactions were associated with behavioural outcomes.

### Testing whether associations between cognitively stimulating parenting and behavioural outcomes vary by group

**Fig. 4:**
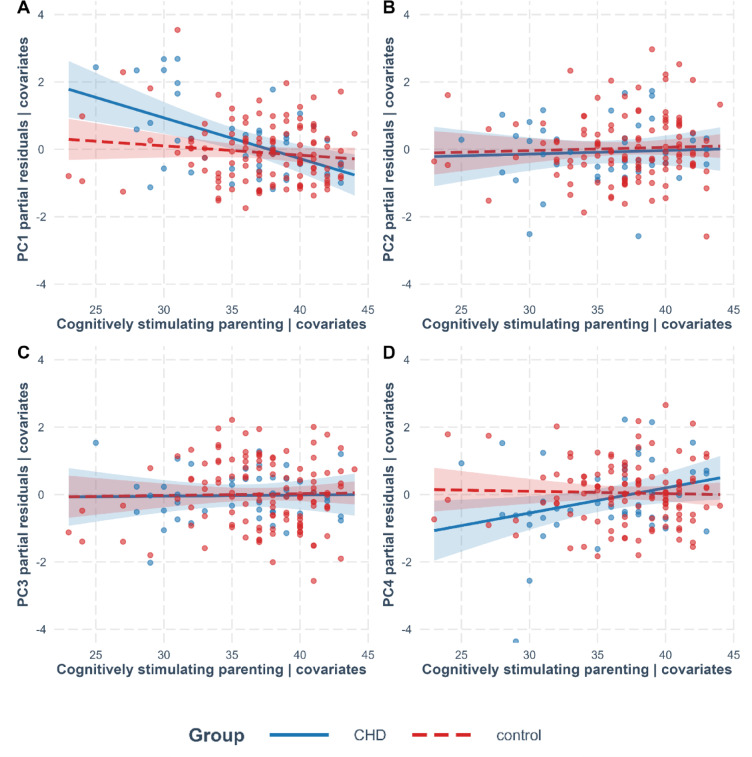
Association between cognitively stimulating parenting and (A) PC1, (B) PC2, (C) PC3, and (D) PC4 in preschool children with CHD (blue) and controls (red), after adjusting for GA at birth, sex, and IMD. Abbreviations: GA, gestational age; IMD, index of multiple deprivation; PC, principal component.

A significant interaction between group and CSPS was observed for the “neurodevelopmental difficulties” component (PC1), after adjusting for GA at birth, sex and IMD (B [95% CI] 0.094 [0.020 to 0.168], t = 2.504, pFDR = 0.043) (Supplementary Table S2). Specifically, a higher CSPS score was associated with fewer neurodevelopmental difficulties in children with CHD, but not in controls (Fig. 4). No other group-CSPS interactions were significant (Supplementary Table S2).

#### **Testing whether associations between neonatal SCNs and behavioural outcomes vary by the level of cognitive stimulation at home**

**Fig. 5:**
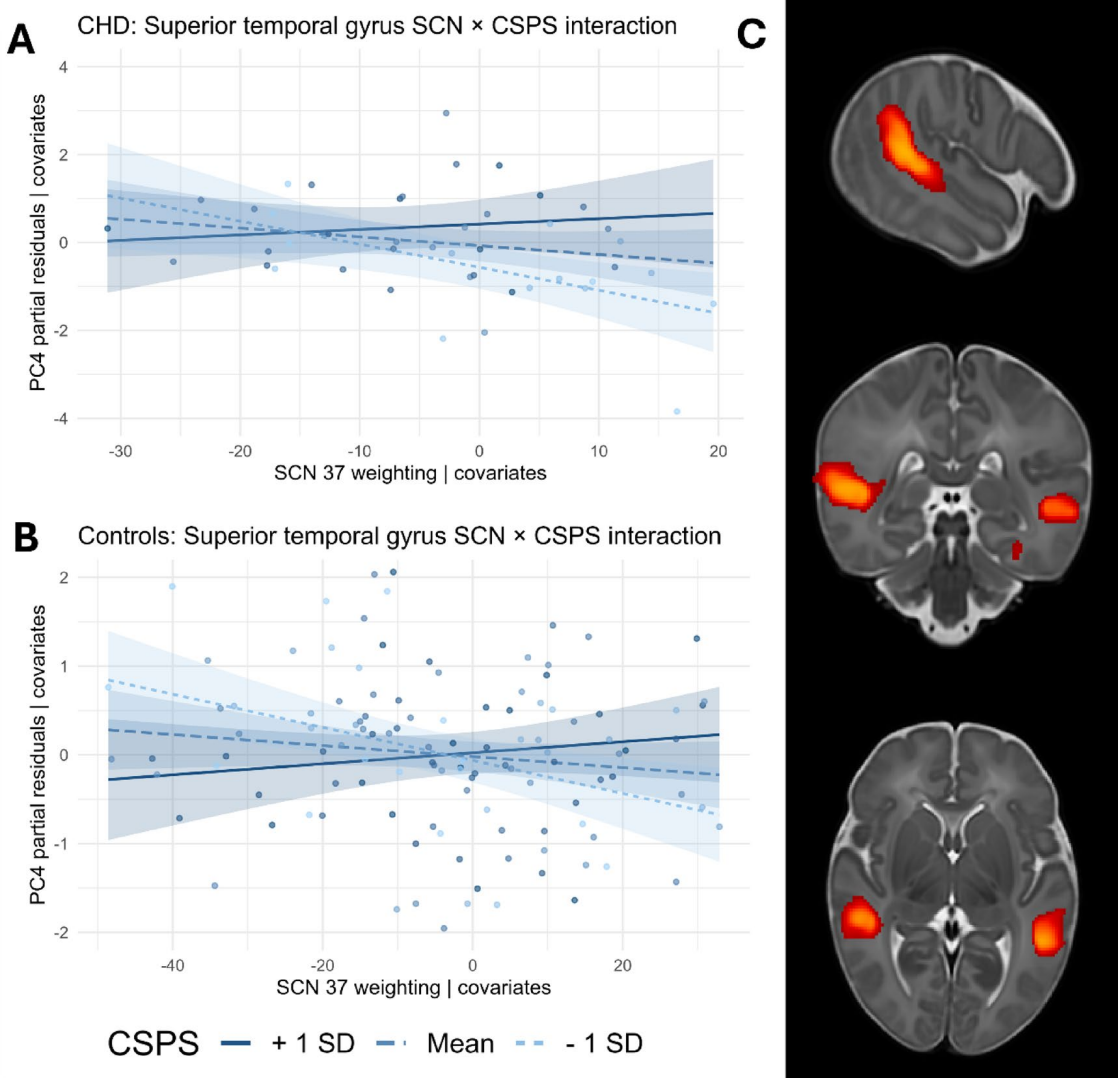
(**A-B**) Cognitively stimulating parenting significantly moderates the association between the superior temporal gyrus SCN (SCN 37) and empathy (PC4) in childhood, after adjusting for GA at birth, PMA at scan, sex, and IMD, in children with CHD and in controls. (**C**) Visualization of SCN 37. Abbreviations: GA, gestational age; CSPS, cognitively stimulating parenting scale; IMD, index of multiple deprivation; PC, principal component; PMA, postmenstrual age; SCN, structural covariance network.

A significant interaction between CSPS and the superior temporal gyrus SCN (SCN 37) was observed for the “empathy” component (PC4) in both children with CHD (B [95% CI] 0.007 [0.001 to 0.013], t = 3.418, pFDR = 0.048) (Fig. 5A; Supplementary Table S6) and in control children (0.003 [0.001 to 0.005], t = 3.390, pFDR = 0.047) (Fig. 5B; Supplementary Table S6).

No significant three-way interaction (SCN x CSPS x group) was observed for the superior temporal gyrus SCN (SCN 37) (B [95% CI] -0.001 [-0.007 to 0.005], t = -0.36, *p* = 0.714), indicating no evidence that this moderation effect differed between groups.

**Fig. 6:**
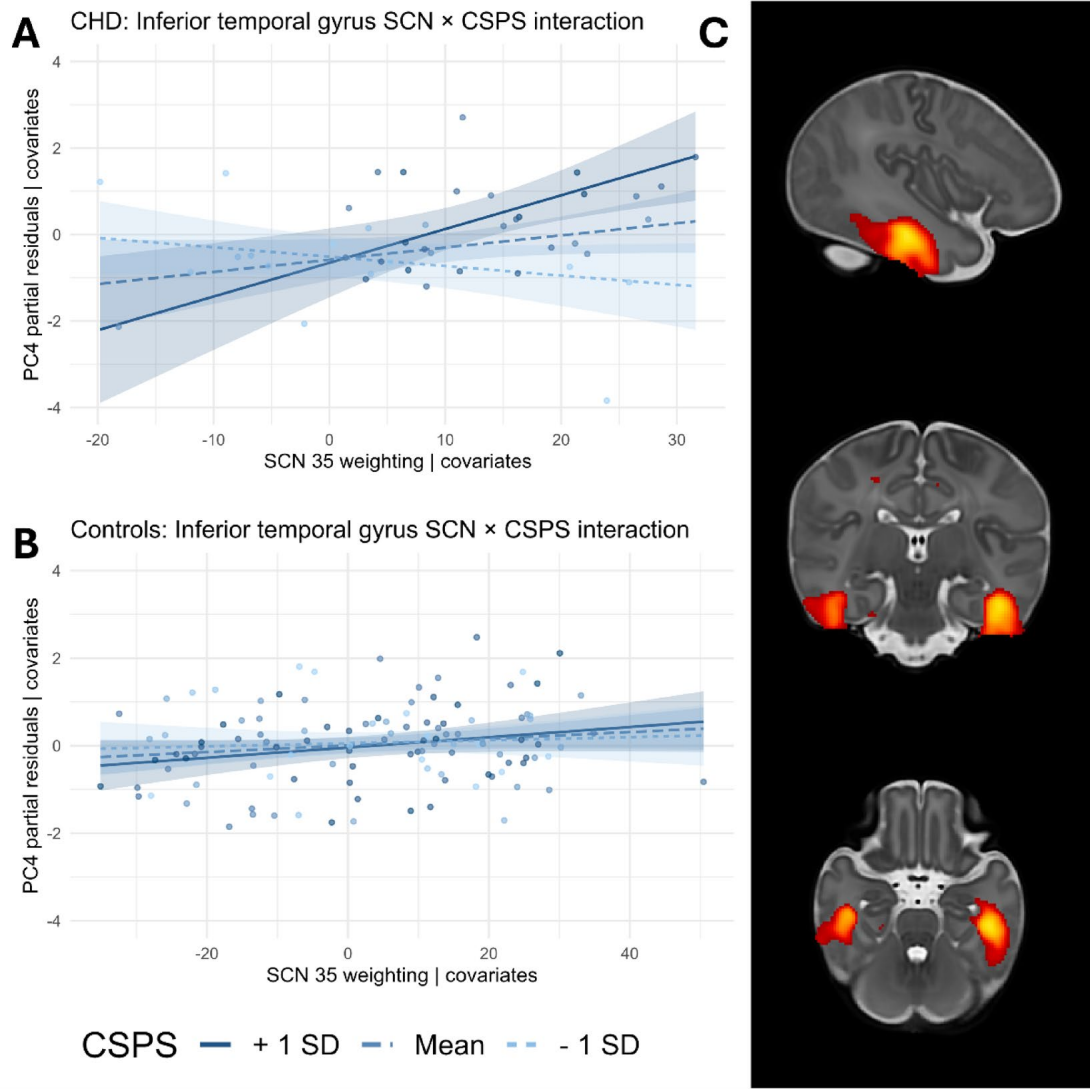
Cognitively stimulating parenting significantly moderates the association between the anterior inferior temporal gyrus SCN (SCN 35) and empathy (PC4) in childhood, after adjusting for GA at birth, PMA at scan, sex, and IMD, in (**A**) children with CHD, (**B**) but not in controls. (**C**) Visualization of SCN 35. Abbreviations: GA, gestational age; CSPS, cognitively stimulating parenting scale; IMD, index of multiple deprivation; PC, principal component; PMA, postmenstrual age; SCN, structural covariance network.

A significant interaction between CSPS and the anterior inferior temporal gyrus SCN (SCN 35) was observed for the “empathy” component (PC4) in children with CHD (B [95% CI] 0.011 [0.005 to 0.016], t = 3.720, pFDR = 0.040) (Fig. 6A; Supplementary Table S6). This relationship was not significant in controls (0.001 [-0.001 to 0.002], t = 0.797, pFDR = 0.814) (Fig. 6B; Supplementary Table S6). No SCN-CSPS interactions were significantly associated with PC1, PC2 or PC3 (Supplementary Tables S3-5).

A significant three-way interaction (SCN x CSPS x group) was observed for the anterior inferior temporal gyrus SCN (SCN 35) (B [95% CI] -0.006 [-0.001 to -0.003], t = -2.1, *p* = 0.035), indicating that the association between SCN 35 and the “empathy” component (PC4) was differentially moderated by cognitively stimulating parenting in children with CHD compared to controls.

### Sensitivity analyses

Additional sensitivity analyses were performed, including maternal education as a covariate. The interaction between group and the SCN encompassing the anterior thalamus and cingulum (SCN 30) (B [95% CI] -0.068 [-0.115 to -0.020], t = -2.80, *p* = 0.004), and between group and CSPS (0.094 [0.016 to 0.172], t = 2.39, *p* = 0.017), remained significantly associated with the “empathy” component (PC4) and the “neurodevelopmental difficulties” component (PC1), respectively.

Similarly, the interaction between CSPS and the anterior inferior temporal gyrus SCN (SCN 35) remained significantly associated with the “empathy” component (PC4) in children with CHD (B [95% CI] 0.009 [0.0004 to 0.014], t = 3.87, *p* = 0.001) but not in control children (0.002 [-0.0003 to 0.005], t = 1.71, *p* = 0.095), after adjusting for maternal education.

In contrast, the interaction between CSPS and the superior temporal gyrus SCN (SCN 37) was no longer significantly associated with the “empathy” component (PC4) in children with CHD (B [95% CI] 0.005 [-0.0011 to 0.0101], t = 1.65, *p* = 0.102) or in control children (0.0026 [0.00002 to 0.0052], t = 1.99, *p* = 0.055) after adjusting for maternal education.

## Discussion

In this study, we investigated the relationship between neonatal brain structure, cognitively stimulating parenting and behavioural outcomes in children with CHD and control children. Using PCA, we identified four components representing distinct behavioural dimensions: executive function deficits, ADHD symptoms and autistic traits (i.e., neurodevelopmental difficulties; PC1), emotional dysregulation (PC2), social function (PC3), and empathy (PC4). Our findings demonstrate that in preschool children with CHD, neonatal morphometry of the anterior thalamus and cingulum was associated with empathy, a relationship not observed in control children. Furthermore, in children with CHD, greater cognitively stimulating parenting was associated with fewer neurodevelopmental difficulties (i.e., executive function deficits, ADHD symptoms and autistic traits). We also demonstrate that cognitively stimulating parenting moderated the relationship between neonatal brain structure and empathy in childhood.

Previous studies have highlighted the role of subcortical brain volumes in neurodevelopmental outcomes in individuals with CHD across the lifespan^15,25,50–52^. The thalamus, in particular, appears to be especially vulnerable to altered development. Smaller postoperative volumes of the thalamus have been associated with lower intelligence quotient scores at 6 years in children with CHD^25^. We have previously shown that infants with CHD have smaller thalamic volumes^16^, and that neonatal thalamic volume predicts cognitive performance at 22 months in infants with CHD^15^. In the present study, we extend these findings by identifying an association between anterior thalamic and cingulum morphometry and empathy in preschool children with CHD. The anterior thalamus and cingulum form key components of the limbic circuitry^53,54^, which support sensory, emotional, and cognitive processes. Evidence from deep brain stimulation studies in adults implicates the anterior thalamic nuclei in emotion processing^55,56^. Moreover, lesion studies support the role of the anterior cingulate in empathy^57,58^. Empathy serves as a cornerstone of socio-emotional development, underpinning the capacity to form meaningful social relationships, regulate affect and engage in prosocial behaviour. Differences in early limbic circuitry may be associated with socio-emotional vulnerabilities in children with CHD, contributing to the early socio-emotional difficulties that have been documented in toddlers^27,59^ and children^60–62^ with CHD.

Our previous study using the same SCN analysis approach highlighted differences in neonatal brain morphometry between infants with CHD and control infants, including in deep grey matter structures, the cingulate, corpus callosum and CSF spaces^38^. However, this previous study did not identify significant associations between neonatal brain morphometry and cognitive or motor outcomes at 22 months in infants with CHD^38^. It is possible that this difference may reflect developmental timing, such that the association between neonatal brain morphology, as identified using SCN analysis, and neurodevelopmental outcomes may become more evident in older children with CHD. The emergence of more complex socio-emotional capabilities, such as understanding and responding to others’ feelings, which are captured by parent-reported measures such as the Empathy Questionnaire, continues to emerge across the preschool years and beyond^63^.

Our finding that cognitively stimulating parenting was associated with fewer neurodevelopmental difficulties (i.e., executive function deficits, attentional difficulties and autistic traits) in children with CHD but not in controls is consistent with the hypothesis that an enriched home environment may promote resilient development within at-risk populations^64^. These findings build on our previous work demonstrating associations between a cognitively stimulating home environment and improved cognition in toddlers with CHD^45^, and better executive function^46^ and fewer ADHD symptoms and peer relationship problems^47^ in preschool children with CHD, but not in healthy preschool children^46,47^. Similar benefits of an enriched home environment have been observed in preterm children^36,65^, and in children with hypoxic-ischaemic encephalopathy^66^. The mechanisms by which a more stimulating home environment supports improved outcomes in at-risk populations are not clear, but rodent models of neonatal brain injury demonstrate that environmental enrichment can prevent brain tissue loss^67–69^ and promote oligodendrogenesis and myelination following brain injury^70^, suggesting that cognitive stimulation may drive experience-dependent neural plasticity following early neurological insult.

We also observed that cognitively stimulating parenting moderated associations between the morphometry of the superior temporal gyrus and empathy in both children with CHD and controls. The superior temporal gyrus is a critical component of the “social brain,” involved in social cognition by integrating auditory, visual and motion cues to support face perception, gaze following, perception of movement and mentalizing^71^. We further identified a moderating effect of a cognitively stimulating home environment on the association between anterior inferior temporal gyrus morphometry and empathy in preschool children with CHD. The inferior temporal cortex is integral to the visual processing of socially salient cues such as faces and emotions^72,73^. Together, these findings underscore the importance of the temporal cortex in socio-emotional development and suggest that these regions may be particularly sensitive to environmental factors during childhood.

Relatively few studies have explored the role of socioeconomic factors or the home environment as modifiers of the relationship between neonatal brain structure and outcomes. In preterm infants, socioeconomic status (measured by maternal education) moderated associations between neonatal anterior hippocampal volumes, hippocampal functional connectivity with regions including the superior and inferior temporal gyri, and cognitive outcomes at 18 months^74^. In another study, the association between brain injury and poorer cognitive outcomes in children born preterm was attenuated among those born to mothers with higher educational attainment^75^. In contrast, in a cohort consisting of predominantly full-term infants, neither social disadvantage nor parenting behaviours interacted with neonatal brain volumes to predict cognitive outcomes at 2 years^76^. Although maternal education differed between children with CHD and controls in our study, sensitivity analyses including maternal education as a covariate did not alter the main findings, with the exception of the interaction between the superior temporal gyrus SCN and cognitively stimulating parenting, which was attenuated, suggesting that this association may be partially influenced by maternal education. Taken together with our findings, these studies suggest that socio-environmental factors may moderate the impact of early brain development on neurodevelopmental outcomes in clinical populations. However, it is important to note that neurodevelopmental outcomes are shaped by a range of environmental influences, including factors such as nutrition^77^ and parental mental health^78^. Future research, incorporating additional environmental measures, is needed to determine whether the home environment continues to shape neurodevelopment in children with CHD into later childhood and adolescence.

These findings have important clinical implications. Interventions aimed at enhancing cognitively stimulating parenting and integrating family-centred support into early clinical care pathways could represent feasible and scalable approaches to promote favourable behavioural outcomes in children with CHD; however, this requires further investigation.

This study has some limitations. IMD, based on residential postcode, provides only a neighbourhood-level measure of socioeconomic status and may not fully capture individual income deprivation^79^. We used validated parental questionnaires to assess childhood behaviour. Nevertheless, the use of parental reports could result in underreporting of behavioural problems^80^. The sample sizes between the CHD and control groups were unbalanced. However, permutation-based inference is less sensitive to unequal group sizes, supporting the robustness of the reported findings^81^. Nonetheless, the smaller sample size in the CHD group may have reduced statistical power to detect more subtle effects. Post hoc power analysis indicated that the study was powered to detect small-to-medium effect sizes (partial f^2^ = 0.13) for the primary two-way interaction analyses at 80% power.

## Conclusions

Our findings provide insights into the neurobiological and environmental determinants of behavioural outcomes in preschool children with CHD. We demonstrate that the morphometry of the neonatal anterior thalamus and cingulum is associated with empathy in preschool children with CHD, but not in controls. We also show that a more cognitively stimulating home environment is associated with fewer neurodevelopmental difficulties and that the relationship between neonatal brain structure and empathy is moderated by cognitively stimulating parenting. These results underscore the importance of the early home environment as a modifiable factor in supporting neurodevelopment in children with CHD.

## Methods

### Ethical approval

Neonatal brain MRI data for the CHD group were acquired as part of The Congenital Heart Disease Imaging Programme (CHIP) and for the control group were obtained from the open-access Developing Human Connectome Project (dHCP) neonatal MRI database (www.developingconnectome.org; available at: https://nda.nih.gov/edit_collection.html?id = 3955)^82^. The study was approved by the National Research Ethics Committee (CHIP neuroimaging: 07/H0707/105; dHCP neuroimaging: 14/LO/1169; outcome assessments for both groups; 19/LO/0451). Informed written parental consent was obtained before collection of MRI and behavioural outcome data. All methods were carried out in accordance with relevant guidelines and regulations.

### Participants with CHD

#### Recruitment and CHD categorization

Infants with CHD were prospectively recruited from the Neonatal and Paediatric Intensive Care Units at the Evelina London Children’s Hospital. Participants were recruited if they had critical or serious CHD, as described previously^15^. Each infant was also allocated to one of three diagnostic CHD categories based on the hemodynamic impact of the underlying cardiac diagnosis, using the sequential segmental approach: (1) abnormal streaming of the blood; (2) left-sided cardiac lesions; or (3) right‐sided cardiac lesions^83^.

#### Inclusion criteria

Infants with CHD were eligible for inclusion if they had a diagnosis of critical or serious CHD; if they did not undergo any other neonatal surgery before their cardiac surgery or intervention; if they were born ≥ 36.00 gestational weeks; if they were scanned between 37 and 46 postmenstrual weeks; if they had good quality T2-weighted MRI data and if they had behavioural outcome data at 4–6 years of age. Exclusion criteria were suspected or confirmed chromosomal abnormality/genetic syndrome (Supplementary Fig. S2).

### Control participants

#### Inclusion criteria

Control infants were included if they were born ≥ 36.00 gestational weeks; if they were scanned between 37 and 46 postmenstrual weeks; if they had good quality T2-weighted MRI data; if no major brain lesions (arterial ischemic stroke, parenchymal haemorrhage, > 10 foci of white matter injury) were reported on their neuroimaging after review by two perinatal neuroradiologists, and if they had behavioural outcome data at 4–6 years of age (Supplementary Fig. S2).

#### **Clinical Information**

Birth weight was extracted from clinical notes and converted to Z-scores using the UK-WHO 1990 normative data^84^. Infants were categorised as appropriate for gestational age if birth weight Z-scores were 10th to 90th percentile.

#### **Neonatal MRI Acquisition**

MRI for both infants with CHD and control infants was performed on a Philips Achieva 3 Tesla system (Best, NL) situated on the Neonatal Unit at St Thomas’ Hospital, London, UK, as described previously^15^.

Infants were scanned with a 32-channel neonatal head coil and neonatal positioning system (Rapid Biomedical GmbH, Rimpar DE)^85^. T2-weighted images were acquired using a multi-slice Turbo Spin Echo sequence [repetition time/ echo time = 12000/156 ms; voxel size = 0.8 × 0.8 × 1.6 mm, SENSE factor 2.0 (axial) and 2.5 (sagittal)]. T2-weighted volumes were reconstructed using a dedicated algorithm to correct motion and integrate data from both acquired stacks (reconstructed voxel size = 0.5 mm^3^)^86,87^.

#### **MR Image processing, Image registration and Jacobian determinant computation**

The pipeline utilised for the extraction and analysis of SCNs is summarised in Supplementary Fig. S3. T2-weighted images were processed using the dHCP structural pipeline for bias field correction and brain extraction^88^. Each native subject T2-weighted dataset was registered to a week-specific template and then to the 40-week GA template of the dHCP neonatal atlas (https://gin.g-node.org/BioMedIA/dhcp-volumetric-atlas-groupwise)^89^, using ANTs Symmetric diffeomorphic image registration^90^. The log Jacobian determinant maps of the warp were computed for each infant, representing the contraction and expansion of brain regions. A single 4D set of volumes was generated by concatenating the log Jacobian determinants for the control cohort, and these were used as the input for ICA extraction of networks. Data quality was assessed through visual inspection at multiple stages of preprocessing, including registration steps. No infants were excluded due to poor registration. All imaging data were processed by a single author (B.G) using a previously published pipeline^38^.

### Independent Component Analysis (ICA) of Jacobian determinants

A canonical ICA algorithm, implemented in Python with the nilearn package^90^, was applied to the log Jacobian determinants of the control cohort, as previously reported^37,38^. This algorithm breaks down the input data into maximally independent components (i.e., SCNs) by separating the mixed signals into separate components with non-Gaussian distributions. The number of SCNs was set at *n* = 40 to balance robustness and interpretability, as previously described^37,38,92^.

To quantify the representation of each network in each subject, FSL’s general linear model was applied to the SCN maps and the Jacobians, extracting a weighting for each SCN in each infant (for both CHD and controls), which could be used for subsequent linear regression analyses^81,93^.

###  Behavioural Outcomes

Parents completed the following questionnaires on children’s temperament, behaviour, and executive functioning: the Children’s Behaviour Questionnaire, Very Short Form (CBQ)^94^; the Empathy Questionnaire (EmQue)^95^; the Strengths and Difficulties Questionnaire (SDQ)^96^; the ADHD Rating Scale, Fourth edition (ADHD-RS)^97^; the Social Communications Questionnaire (SCQ)^98^; and the Behaviour Rating Inventory of Executive Function, Preschool version (BRIEF-P)^99^.

### **Cognitively Stimulating Parenting**

Parents completed the Cognitively Stimulating Parenting Scale (CSPS)^45^, a 28-item questionnaire adaptation of the Home Observation for Measurement of the Environment Inventory^100^. The CSPS assesses the availability and variety of experiences that promote cognitive stimulation at home and in the family, as previously described^46,47^. Higher CSPS scores indicate greater levels of cognitive stimulation within the home environment.

### **Socioeconomic status**

Index of multiple deprivation (IMD) was derived from the residence postcode when follow-up questionnaires were posted to parents. IMD is a composite measure of socioeconomic status in England encompassing factors such as income, employment, education, health and crime (http://imd-by-postcode.opendatacommunities.org/). IMD was calculated from the 2015 data release and reported as quintiles.

#### **Maternal education**

Parents were asked to report the highest maternal education level when follow-up questionnaires were posted to parents (six categories: GCSE/O-levels, A-levels, vocational/college education, university undergraduate degree, university master’s degree and university doctorate degree).

#### **Principal component analysis of outcome data**

To summarize the 19 behavioural variables obtained from all six questionnaires, a PCA was performed using oblimin rotation. Before conducting PCA, all scores were regressed against age at the follow-up assessment. Residuals from these regressions were Z-scored and used in the subsequent PCA analysis.

### Statistical analysis

All analyses were conducted in R v.4.3.1 (R Core Team 2023). To facilitate statistical analyses and ensure sufficient sample sizes within each category, maternal education data were grouped into three categories: Below university undergraduate, university undergraduate, and university postgraduate.

A Shapiro-Wilk test was used to assess normality. Demographic variables were presented as medians and interquartile ranges (IQR), means and standard deviation (SD) or counts. Differences in demographics between the CHD cohort and healthy controls were assessed with the Mann-Whitney U test or χ^2^. Differences in CSPS scores between groups were assessed with the Mann‐Whitney U test. Differences in birth weight Z-scores between groups were assessed with a t-test. Differences in IMD quintiles, maternal education and number of participants who were appropriate for gestational age between groups were assessed with Fisher’s exact test.

Multiple linear regressions with permutation testing (*n* = 5,000) were used for all subsequent analyses. Three sets of interaction effects were tested: (i) group × SCN, to assess whether associations between neonatal brain structure and behavioural outcomes existed and if these differed between children with CHD and healthy controls (Eq. 1); (ii) group × CSPS, to assess whether the relationship between home environment and behavioural outcomes differed between children with CHD and healthy controls (Eq. 2); and (iii) SCN × CSPS, to investigate whether the association between neonatal brain structure and behavioural outcomes was moderated by the level of cognitive stimulation in the home environment in children with CHD and healthy controls (Eq. 3).

Covariates were selected a priori based on prior literature demonstrating associations with neonatal brain development and neurodevelopmental outcomes^15,17^. All models were adjusted for GA at birth, sex, and IMD, with PMA at scan included as an additional covariate in group x SCN interaction and SCN x CSPS interaction models. Linear regressions for the SCN x CSPS interaction model were assessed in the CHD and control groups separately. For significant SCN × CSPS interactions, post-hoc three-way interaction analyses (SCN × CSPS × group) were performed to formally test whether the moderation effect differed between groups (Eq. 4).

Additional sensitivity analyses were performed for models with significant interactions, including maternal education as an additional covariate. Permuted p-values were corrected for multiple comparisons using Benjamini–Hochberg False Discovery Rate (FDR)^101^.

(1) 

$$\:{\mathrm{PC}}_{i}\mathrm{\:~\:}{\beta}_{0}\mathrm{+\:}{\beta}_{1}{\mathrm{SCN}}_{i}\mathrm{\:+\:}{\beta}_{2}{\mathrm{Group}}_{i}\mathrm{\:+\:}{\beta}_{3}\mathrm{(}{\mathrm{SCN}}_{i}\mathrm{\:*\:}{\mathrm{Group}}_{i}\mathrm{)\:+\:}{\beta}_{4}{\mathrm{GA\:at\:birth}}_{i}\mathrm{\:+\:}{{\beta}_{5}\mathrm{PMA}\:\mathrm{at\:scan}}_{i}\:\mathrm{+\:}{\beta}_{6}{\mathrm{Sex}}_{i}\mathrm{+\:}{\beta}_{7}{\mathrm{IMD}}_{i\text{}}\mathrm{+\:}{\epsilon\:}_{i}$$

(2) 

$$\:{\mathrm{PC}}_{i}{\: {\sim} \:}{\beta}_{0}\mathrm{+\:}{\beta}_{1}{\mathrm{CSPS}}_{i}\mathrm{\:+\:}{\beta}_{2}{\mathrm{Group}}_{i}\mathrm{\:+\:}{\beta}_{3}\mathrm{(}{\mathrm{CSPS}}_{i}\mathrm{\:*\:}{\mathrm{Group}}_{i}\mathrm{)\:+\:}{\beta}_{4}{\mathrm{GA\:at\:birth}}_{i}\mathrm{\:+\:}{{\beta}_{5}\mathrm{Sex}}_{i}\:\mathrm{+\:}{\beta}_{6}{\mathrm{IMD}}_{i}+\:{\epsilon\:}_{i}$$

(3) 

$$\:{\mathrm{PC}}_{i}{\:{\sim}\:}{\beta}_{\mathrm{0}}\mathrm{+\:}{\beta}_{\mathrm{1}}{\mathrm{SCN}}_{i}\mathrm{\:+\:}{\beta}_{\mathrm{2}}{\mathrm{CSPS}}_{i}\mathrm{\:+\:}{\beta}_{\mathrm{3}}\mathrm{(}{\mathrm{SCN}}_{i}\mathrm{\:*\:}{\mathrm{CSPS}}_{i}\mathrm{)\:+\:}{\beta}_{\mathrm{4}}{\mathrm{GA\:at\:birth}}_{i}\mathrm{\:+\:}{{\beta}_{\mathrm{5}}\mathrm{PMA}\mathrm{\:at\:scan}}_{i}\mathrm{\:+\:}{\beta}_{\mathrm{6}}{\mathrm{Sex}}_{i}\text{}\mathrm{+\:}{\beta}_{\mathrm{7}}{\mathrm{IMD}}_{i\text{}}\mathrm{+\:}{\epsilon\:}_{i}$$

(4) 

$$\:{\mathrm{PC}}_{i}{\:{\sim}\:}{\beta}_{\mathrm{0}}\mathrm{+\:}{\beta}_{\mathrm{1}}{\mathrm{SCN}}_{i}\mathrm{\:+\:}{\beta}_{\mathrm{2}}{\mathrm{CSPS}}_{i}\mathrm{\:+\:}{\beta}_{\mathrm{3}}{\mathrm{Group}}_{i}\text{}\mathrm{+\:}{\beta}_{\mathrm{4}}\mathrm{(}{\mathrm{SCN}}_{i}\mathrm{\:*\:}{\mathrm{CSPS}}_{i}\mathrm{)\:+\:}{\beta}_{\mathrm{5}}\mathrm{(}{\mathrm{SCN}}_{i}\mathrm{\:*}\text{}{\mathrm{Group}}_{i}\mathrm{)\:}\mathrm{+\:}{\beta}_{\mathrm{6}}\mathrm{(}{\mathrm{CSPS}}_{i}\mathrm{\:*\:}{\mathrm{Group}}_{i}\mathrm{)\:}\mathrm{+\:}{\beta}_{\mathrm{7}}$$

$$\:\mathrm{(}{\mathrm{SCN}}_{i}\mathrm{\:*\:}{\mathrm{CSPS}}_{i}\mathrm{\:*\:}{\mathrm{Group}}_{i}\mathrm{)\:}\mathrm{+\:}{\beta}_{\mathrm{8}}{\mathrm{GA\:at\:birth}}_{i}\mathrm{\:+\:}{{\beta}_{\mathrm{9}}\mathrm{PMA}\mathrm{\:at\:scan}}_{i}\mathrm{\:+\:}{\beta}_{\mathrm{10}}{\mathrm{Sex}}_{i}\text{}\mathrm{+\:}{\beta}_{\mathrm{11}}{\mathrm{IMD}}_{i\text{}}\mathrm{+\:}{\epsilon\:}_{i}$$

## Supplementary Information

Below is the link to the electronic supplementary material.

Supplementary Material 1

## Data Availability

The data that support the findings of this study are available from the corresponding author upon reasonable request.
