# Supplementary material for: Neonatal brain structure, cognitively stimulating parenting and behavioural outcomes in preschool children with congenital heart disease and controls
Source: Sci Rep. 2026 May 29;16:24636. doi: 10.1038/s41598-026-55283-5 (PMC13454275; doi:10.1038/s41598-026-55283-5)
Supplement: Supplementary file 1 [file 41598_2026_55283_MOESM1_ESM.docx]

**Supplementary Materials**

**Neonatal brain structure, cognitively stimulating parenting and behavioural outcomes in preschool children with congenital heart disease and controls**

Barat Gal-Er^1^, Alexandra F. Bonthrone^1^, Andrew T.M. Chew^1^, Sian Wilson^1,2^, Daniel Cromb^1^, Alexia Egloff^1^, Kuberan Pushparajah^3,4^, John Simpson^3,4^, Mirthe E.M. van der Meijden^1^, Mary A. Rutherford^1^, Joseph V. Hajnal^1,5^, A. David Edwards^1^, Jonathan O’Muircheartaigh^1,6,7^, Chiara Nosarti^1,8^, Serena J. Counsell^1*^

1. Centre for the Developing Brain, Research Department of Early Life Imaging, School of Biomedical Engineering and Imaging Sciences, King’s College London, London, UK

2. Department of Computer Science, University of Oxford, Oxford, UK

3. Department of Cardiovascular Imaging, School of Biomedical Engineering and Imaging Sciences, King’s College London, London, UK

4. Department of Fetal and Paediatric Cardiology, Evelina London Children’s Hospital, London, UK

5. Research Department of Imaging Physics & Engineering, School of Biomedical Engineering and Imaging Sciences, King's College London, London, UK

6. Department for Forensic and Neurodevelopmental Sciences, Institute of Psychiatry, Psychology and Neuroscience, King’s College London, London, UK

7. MRC Centre for Neurodevelopmental Disorders, King's College London, London, UK

8. Department of Child and Adolescent Psychiatry, Institute of Psychiatry, Psychology and Neuroscience, King's College London, London, UK

**Supplementary Figure 1.** Scree plot showing the variance explained [eigenvalues] (y-axis) by each principal component (x-axis). The horizontal red line shows the number of components retained (k = 4), which was determined by Horn’s parallel analysis.

**Supplementary Figure 2.** Flowchart of participant inclusion for both the CHD and control groups.

**Supplementary Figure 3.** Schematic overview of the analysis pipeline. A) T2w neonatal MRI was registered to the dHCP 40-week atlas, using non-linear registration. B) The log Jacobian determinant of the non-linear warp was computed. C) Canonical independent component analysis was applied to the Jacobian determinant images of the control cohort to extract 40 SCNs. D) SCN weightings were extracted for each SCN for each subject in both control and CHD groups. E) Principal component analysis was performed to summarise the 19 behavioural measures obtained from six questionnaires, and four components were retained. F) Multiple linear regressions with permutation testing (n = 5,000) were used to test three sets of interaction effects. Abbreviations: CHD, congenital heart disease; CSPS, cognitively stimulating parenting scale; GA, gestational age; IMD, index of multiple deprivation; PC, principal component; PMA, postmenstrual age; SCN, structural covariance network.

**Supplementary Table S1.** Moderating effect of group on the association between neonatal SCNs and behavioural outcomes in childhood (PC1-4).

| **SCN** | **B (95% CI)** | **t-score** | **pFDR** |
| --- | --- | --- | --- |
| **PC1** | | | |
| (1) CSF around anterior temporal lobe | <0.001  (-0.016, 0.016) | 0.036 | 0.997 |
| (2) Anterior temporal region | -0.009  (-0.035, 0.017) | -0.676 | 0.967 |
| (3) Caudate + lentiform nucleus | 0.016  (-0.013, 0.045) | 1.074 | 0.690 |
| (4) Temporal operculum + anterior frontal WM | -0.008  (-0.044, 0.028) | -0.443 | 0.967 |
| (5) Posterior lateral ventricles | 0.018  (0.001, 0.036) | 2.041 | 0.169 |
| (6) Anterior frontal region | 0.032  (0.008, 0.056) | 2.680 | 0.169 |
| (7) Superior cerebellum, pons, CSF around hippocampus | <0.001  (-0.032, 0.032) | 0.018 | 0.997 |
| (8) Medial cerebellum | -0.013  (-0.040, 0.014) | -0.979 | 0.757 |
| (9) Left visual cortex | 0.002  (-0.026, 0.029) | 0.113 | 0.976 |
| (10) Cerebellar CSF | 0.005  (-0.019, 0.029) | 0.418 | 0.967 |
| (11) Cerebellum + brainstem | 0.048  (0.008, 0.088) | 2.347 | 0.169 |
| (12) Frontal WM region | -0.009  (-0.032, 0.014) | -0.789 | 0.868 |
| (13) Medial motor cortex | 0.016  (-0.008, 0.039) | 1.335 | 0.630 |
| (14) Occipito-cerebellar CSF + splenium | 0.010  (-0.014, 0.034) | 0.790 | 0.861 |
| (15) Anterior cingulate | -0.012  (-0.032, 0.007) | -1.236 | 0.630 |
| (16) Medial occipital region | -0.001  (-0.016, 0.014) | -0.172 | 0.967 |
| (17) Somatosensory cortex | -0.010  (-0.030, 0.011) | -0.941 | 0.761 |
| (18) Parietal-occipital region | -0.002  (-0.019, 0.016) | -0.180 | 0.967 |
| (19) Anterior lateral ventricles | 0.006  (-0.013, 0.026) | 0.628 | 0.967 |
| (20) Posterior thalamus + hippocampus | -0.013  (-0.043, 0.017) | -0.858 | 0.805 |
| (21) CSF around insula | -0.019  (-0.039, 0.001) | -1.848 | 0.369 |
| (22) Inferior frontal region | -0.012  (-0.041, 0.018) | -0.786 | 0.859 |
| (23) Motor cortex | -0.010  (-0.043, 0.023) | -0.611 | 0.967 |
| (24) Frontal midline (left) | -0.023  (-0.052, 0.006) | -1.593 | 0.531 |
| (25) Inferior frontal gyrus | -0.008  (-0.031, 0.015) | -0.646 | 0.967 |
| (26) Insula | -0.011  (-0.037, 0.015) | -0.808 | 0.828 |
| (27) Orbitofrontal region (right) | 0.009  (-0.021, 0.038) | 0.588 | 0.967 |
| (28) Anterior infratentorial CSF + orbitofrontal CSF | 0.009  (-0.029, 0.047) | 0.471 | 0.967 |
| (29) Superior frontal CSF | -0.021  (-0.046, 0.004) | -1.626 | 0.526 |
| (30) Anterior thalamus + cingulum | 0.004  (-0.038, 0.047) | 0.198 | 0.967 |
| (31) Infratentorial CSF | -0.003  (-0.031, 0.026) | -0.174 | 0.967 |
| (32) Fusiform gyrus | -0.010  (-0.041, 0.021) | -0.627 | 0.967 |
| (33) Inferior temporal gyrus – posterior (left) | -0.016  (-0.046, 0.013) | -1.093 | 0.672 |
| (34) Middle frontal gyrus | 0.023  (-0.010, 0.055) | 1.384 | 0.625 |
| (35) Inferior temporal gyrus – anterior | 0.022  (-0.004, 0.047) | 1.672 | 0.516 |
| (36) Posterior parietal region | 0.001  (-0.021, 0.023) | 0.069 | 0.988 |
| (37) Superior temporal gyrus | -0.013  (-0.040, 0.014) | -0.942 | 0.757 |
| (38) Precuneus | -0.006  (-0.024, 0.011) | -0.710 | 0.930 |
| (39) Inferior temporal gyrus – posterior (right) | -0.007  (-0.031, 0.017) | -0.580 | 0.967 |
| (40) Periventricular crossroads | 0.013  (-0.005, 0.032) | 1.403 | 0.625 |
| **PC2** | | | |
| (1) CSF around anterior temporal lobe | 0.006  (-0.010, 0.022) | 0.778 | 0.542 |
| (2) Anterior temporal region | -0.014  (-0.040, 0.011) | -1.110 | 0.381 |
| (3) Caudate + lentiform nucleus | -0.008  (-0.037, 0.021) | -0.555 | 0.677 |
| (4) Temporal operculum + anterior frontal WM | 0.014  (-0.021, 0.050) | 0.798 | 0.530 |
| (5) Posterior lateral ventricles | 0.006  (-0.012, 0.024) | 0.661 | 0.610 |
| (6) Anterior frontal region | 0.005  (-0.019, 0.028) | 0.393 | 0.748 |
| (7) Superior cerebellum, pons, CSF around hippocampus | -0.009  (-0.041, 0.023) | -0.551 | 0.656 |
| (8) Medial cerebellum | -0.008  (-0.035, 0.019) | -0.609 | 0.643 |
| (9) Left visual cortex | -0.017  (-0.044, 0.010) | -1.243 | 0.357 |
| (10) Cerebellar CSF | -0.004  (-0.027, 0.020) | -0.323 | 0.802 |
| (11) Cerebellum + brainstem | 0.021  (-0.020, 0.061) | 1.023 | 0.407 |
| (12) Frontal WM region | -0.005  (-0.027, 0.017) | -0.452 | 0.728 |
| (13) Medial motor cortex | -0.016  (-0.039, 0.007) | -1.389 | 0.337 |
| (14) Occipito-cerebellar CSF + splenium | -0.009  (-0.033, 0.015) | -0.756 | 0.546 |
| (15) Anterior cingulate | -0.010  (-0.030, 0.009) | -1.034 | 0.406 |
| (16) Medial occipital region | <0.001  (-0.015, 0.015) | -0.025 | 0.983 |
| (17) Somatosensory cortex | -0.019  (-0.040, 0.001) | -1.873 | 0.317 |
| (18) Parietal-occipital region | 0.004  (-0.013, 0.022) | 0.484 | 0.702 |
| (19) Anterior lateral ventricles | 0.010  (-0.010, 0.029) | 1.006 | 0.418 |
| (20) Posterior thalamus + hippocampus | -0.008  (-0.038, 0.022) | -0.534 | 0.682 |
| (21) CSF around insula | -0.013  (-0.033, 0.007) | -1.284 | 0.346 |
| (22) Inferior frontal region | -0.001  (-0.030, 0.028) | -0.062 | 0.963 |
| (23) Motor cortex | 0.003  (-0.030, 0.036) | 0.208 | 0.859 |
| (24) Frontal midline (left) | -0.031  (-0.059, -0.002) | -2.106 | 0.117 |
| (25) Inferior frontal gyrus | 0.011  (-0.012, 0.034) | 0.929 | 0.450 |
| (26) Insula | 0.013  (-0.013, 0.039) | 0.972 | 0.428 |
| (27) Orbitofrontal region (right) | 0.006  (-0.023, 0.035) | 0.426 | 0.742 |
| (28) Anterior infratentorial CSF + orbitofrontal CSF | -0.009  (-0.047, 0.029) | -0.489 | 0.703 |
| (29) Superior frontal CSF | -0.005  (-0.031, 0.020) | -0.416 | 0.742 |
| (30) Anterior thalamus + cingulum | -0.018  (-0.060, 0.024) | -0.826 | 0.520 |
| (31) Infratentorial CSF | <0.001  (-0.029, 0.029) | 0.011 | 0.990 |
| (32) Fusiform gyrus | 0.014  (-0.016, 0.045) | 0.917 | 0.458 |
| (33) Inferior temporal gyrus – posterior (left) | -0.006  (-0.035, 0.023) | -0.415 | 0.742 |
| (34) Middle frontal gyrus | -0.016  (-0.048, 0.016) | -0.968 | 0.427 |
| (35) Inferior temporal gyrus – anterior | -0.026  (-0.052, -0.001) | -2.041 | 0.317 |
| (36) Posterior parietal region | 0.021  (-0.001, 0.042) | 1.859 | 0.317 |
| (37) Superior temporal gyrus | -0.003  (-0.030, 0.024) | -0.233 | 0.853 |
| (38) Precuneus | 0.005  (-0.012, 0.022) | 0.600 | 0.648 |
| (39) Inferior temporal gyrus – posterior (right) | -0.007  (-0.031, 0.017) | -0.570 | 0.660 |
| (40) Periventricular crossroads | 0.012  (-0.007, 0.030) | 1.208 | 0.362 |
| **PC3** | | | |
| (1) CSF around anterior temporal lobe | 0.002  (-0.013, 0.017) | 0.260 | 0.890 |
| (2) Anterior temporal region | 0.004  (-0.021, 0.029) | 0.331 | 0.864 |
| (3) Caudate + lentiform nucleus | 0.007  (-0.020, 0.035) | 0.528 | 0.836 |
| (4) Temporal operculum + anterior frontal WM | 0.011  (-0.023, 0.046) | 0.645 | 0.804 |
| (5) Posterior lateral ventricles | 0.002  (-0.015, 0.019) | 0.268 | 0.885 |
| (6) Anterior frontal region | -0.001  (-0.024, 0.022) | -0.054 | 0.979 |
| (7) Superior cerebellum, pons, CSF around hippocampus | 0.002  (-0.029, 0.032) | 0.104 | 0.949 |
| (8) Medial cerebellum | -0.004  (-0.030, 0.021) | -0.320 | 0.864 |
| (9) Left visual cortex | 0.001  (-0.025, 0.028) | 0.112 | 0.943 |
| (10) Cerebellar CSF | 0.007  (-0.016, 0.030) | 0.605 | 0.816 |
| (11) Cerebellum + brainstem | -0.006  (-0.046, 0.033) | -0.317 | 0.864 |
| (12) Frontal WM region | 0.014  (-0.007, 0.036) | 1.319 | 0.454 |
| (13) Medial motor cortex | -0.014  (-0.036, 0.008) | -1.237 | 0.468 |
| (14) Occipito-cerebellar CSF + splenium | -0.004  (-0.027, 0.018) | -0.388 | 0.852 |
| (15) Anterior cingulate | -0.003  (-0.022, 0.016) | -0.274 | 0.885 |
| (16) Medial occipital region | -0.004  (-0.019, 0.010) | -0.556 | 0.823 |
| (17) Somatosensory cortex | -0.012  (-0.032, 0.008) | -1.194 | 0.473 |
| (18) Parietal-occipital region | -0.011  (-0.027, 0.006) | -1.308 | 0.458 |
| (19) Anterior lateral ventricles | 0.015  (-0.004, 0.033) | 1.547 | 0.309 |
| (20) Posterior thalamus + hippocampus | -0.003  (-0.032, 0.026) | -0.210 | 0.913 |
| (21) CSF around insula | 0.010  (-0.009, 0.030) | 1.026 | 0.571 |
| (22) Inferior frontal region | -0.011  (-0.039, 0.017 | -0.770 | 0.733 |
| (23) Motor cortex | -0.011  (-0.043, 0.021) | -0.663 | 0.796 |
| (24) Frontal midline (left) | -0.001  (-0.030, 0.027) | -0.095 | 0.959 |
| (25) Inferior frontal gyrus | -0.015  (-0.037**,** 0.007) | -1.349 | 0.431 |
| (26) Insula | -0.010  (-0.035, 0.015) | -0.777 | 0.719 |
| (27) Orbitofrontal region (right) | -0.003  (-0.031, 0.026) | -0.175 | 0.918 |
| (28) Anterior infratentorial CSF + orbitofrontal CSF | 0.037  (0.002, 0.072) | 2.062 | 0.175 |
| (29) Superior frontal CSF | 0.005  (-0.019, 0.029) | 0.437 | 0.852 |
| (30) Anterior thalamus + cingulum | 0.003  (-0.038, 0.043) | 0.134 | 0.943 |
| (31) Infratentorial CSF | <0.001  (-0.028, 0.027) | -0.024 | 0.992 |
| (32) Fusiform gyrus | -0.004  (-0.034, 0.026) | -0.269 | 0.885 |
| (33) Inferior temporal gyrus – posterior (left) | 0.010  (-0.019, 0.038) | 0.672 | 0.780 |
| (34) Middle frontal gyrus | 0.003  (-0.028, 0.034) | 0.179 | 0.918 |
| (35) Inferior temporal gyrus – anterior | 0.020  (-0.005, 0.045) | 1.588 | 0.309 |
| (36) Posterior parietal region | -0.011  (-0.032, 0.011) | -1.000 | 0.571 |
| (37) Superior temporal gyrus | 0.026  (0.001, 0.052) | 2.031 | 0.175 |
| (38) Precuneus | -0.002  (-0.018, 0.015) | -0.216 | 0.897 |
| (39) Inferior temporal gyrus – posterior (right) | 0.001  (-0.021, 0.024) | 0.125 | 0.943 |
| (40) Periventricular crossroads | <0.001  (-0.018, 0.019) | 0.026 | 0.992 |
| **PC4** | | | |
| (1) CSF around anterior temporal lobe | -0.002  (-0.018, 0.015) | -0.211 | 0.941 |
| (2) Anterior temporal region | -0.019  (-0.045, 0.008) | -1.396 | 0.570 |
| (3) Caudate + lentiform nucleus | -0.021  (-0.051, 0.009) | -1.402 | 0.550 |
| (4) Temporal operculum + anterior frontal WM | -0.026  (-0.062, 0.011) | -1.374 | 0.570 |
| (5) Posterior lateral ventricles | 0.011  (-0.007, 0.029) | 1.198 | 0.570 |
| (6) Anterior frontal region | 0.012  (-0.013, 0.036) | 0.946 | 0.612 |
| (7) Superior cerebellum, pons, CSF around hippocampus | 0.027  (-0.006, 0.060) | 1.642 | 0.475 |
| (8) Medial cerebellum | -0.013  (-0.041, 0.015) | -0.922 | 0.616 |
| (9) Left visual cortex | 0.009  (-0.019, 0.037) | 0.631 | 0.748 |
| (10) Cerebellar CSF | -0.016  (-0.040, 0.008) | -1.295 | 0.595 |
| (11) Cerebellum + brainstem | 0.003  (-0.039, 0.044) | 0.118 | 0.966 |
| (12) Frontal WM region | 0.010  (-0.013, 0.033) | 0.868 | 0.673 |
| (13) Medial motor cortex | -0.009  (-0.033, 0.016) | -0.703 | 0.748 |
| (14) Occipito-cerebellar CSF + splenium | 0.008  (-0.017, 0.032) | 0.625 | 0.748 |
| (15) Anterior cingulate | -0.003  (-0.023, 0.018) | -0.271 | 0.938 |
| (16) Medial occipital region | 0.011  (-0.005, 0.026) | 1.374 | 0.570 |
| (17) Somatosensory cortex | -0.010  (-0.031, 0.011) | -0.903 | 0.648 |
| (18) Parietal-occipital region | -0.010  (-0.028, 0.007) | -1.156 | 0.570 |
| (19) Anterior lateral ventricles | 0.012  (-0.008, 0.032) | 1.219 | 0.648 |
| (20) Posterior thalamus + hippocampus | -0.020  (-0.050, 0.011) | -1.291 | 0.570 |
| (21) CSF around insula | <0.001  (-0.020, 0.021) | 0.046 | 0.983 |
| (22) Inferior frontal region | -0.024  (-0.054, 0.006) | -1.576 | 0.550 |
| (23) Motor cortex | 0.009  (-0.025, 0.043) | 0.541 | 0.794 |
| (24) Frontal midline (left) | -0.006  (-0.036, 0.024) | -0.386 | 0.862 |
| (25) Inferior frontal gyrus | -0.012  (-0.036, 0.011) | -1.039 | 0.612 |
| (26) Insula | -0.009  (-0.036, 0.017) | -0.698 | 0.748 |
| (27) Orbitofrontal region (right) | -0.007  (-0.038, 0.023) | -0.487 | 0.794 |
| (28) Anterior infratentorial CSF + orbitofrontal CSF | 0.003  (-0.036, 0.042) | 0.15 | 0.950 |
| (29) Superior frontal CSF | 0.027  (0.001, 0.053) | 2.088 | 0.475 |
| (30) Anterior thalamus + cingulum | -0.068  (-0.110, -0.026) | -3.202 | **0.008*** |
| (31) Infratentorial CSF | 0.010  (-0.019, 0.040) | 0.676 | 0.748 |
| (32) Fusiform gyrus | -0.032  (-0.064, -0.001) | -2.046 | 0.175 |
| (33) Inferior temporal gyrus – posterior (left) | 0.028  (-0.002, 0.058) | 1.844 | 0.475 |
| (34) Middle frontal gyrus | <0.001  (-0.033, 0.034) | 0.002 | 0.998 |
| (35) Inferior temporal gyrus – anterior | -0.003  (-0.030, 0.023) | -0.237 | 0.941 |
| (36) Posterior parietal region | -0.002  (-0.025, 0.021) | -0.18 | 0.950 |
| (37) Superior temporal gyrus | 0.020  (-0.008, 0.047) | 1.428 | 0.550 |
| (38) Precuneus | -0.007  (-0.024, 0.011) | -0.73 | 0.694 |
| (39) Inferior temporal gyrus – posterior (right) | 0.027  (0.003, 0.051) | 2.254 | 0.404 |
| (40) Periventricular crossroads | -0.007  (-0.027, 0.012) | -0.729 | 0.673 |

*Significant results pFDR <0.05; linear regression with 5,000 permutations, model covariates included gestational age at birth, postmenstrual age at scan, sex and index of multiple deprivation. Abbreviations: FDR, false discovery rate; PC, principal component; SCN, structural covariance network.

**Supplementary Table S2.** Moderating effect of group on the association between cognitively stimulating parenting and behavioural outcomes in childhood (PC1-4).

| **Predictor** | **B (95% CI)** | **t-score** | **pFDR** |
| --- | --- | --- | --- |
| **PC1** | | | |
| IMD | -1.26 x 10^-5^ (-3.02 x 10^-5^, 4.96 x 10^-6^) | -1.418 | 0.426 |
| GA at birth | 0.017 (-0.111, 0.145) | 0.260 | 0.796 |
| Sex | -0.186 (-0.482, 0.110) | -1.241 | 0.271 |
| Group | -3.642 (-6.336, -0.948) | -2.670 | **0.041*** |
| CSPS | -0.121 (-0.183, -0.059) | -3.881 | **0.001*** |
| Group x CSPS | 0.094 (0.020, 0.168) | 2.504 | **0.043*** |
| **PC2** | | | |
| IMD | 1.11 x 10^-5^ (-7.42 x 10^-6^, 2.95 x 10^-5^) | 1.183 | 0.426 |
| GA at birth | 0.098 (-0.036, 0.233) | 1.441 | 0.404 |
| Sex | 0.286 (-0.026, 0.597) | 1.811 | 0.148 |
| Group | 0.130 (-2.702, 2.963) | 0.091 | 0.976 |
| CSPS | 0.011 (-0.054, 0.075) | 0.321 | 0.923 |
| Group x CSPS | -0.001 (-0.079, 0.077) | -0.024 | 0.983 |
| **PC3** | | | |
| IMD | -9.05 x 10^-6^ (-2.70 x 10^-5^, 8.89 x 10^-6^ ) | -0.996 | 0.426 |
| GA at birth | -0.084 (-0.214, 0.047) | -1.268 | 0.404 |
| Sex | 0.575 (0.273, 0.877) | 3.756 | **0.002*** |
| Group | -0.042 (-2.791, 2.708) | -0.030 | 0.976 |
| CSPS | 0.003 (-0.060, 0.066) | 0.103 | 0.923 |
| Group x CSPS | 0.002 (-0.073, 0.077) | 0.055 | 0.983 |
| **PC4** | | | |
| IMD | -1.48 x 10^-6^ (-2.01 x 10^-5^, 1.72 x 10^-5^) | -0.157 | 0.878 |
| GA at birth | -0.042 (-0.178, 0.094) | -0.615 | 0.732 |
| Sex | -0.068 (-0.383, 0.246) | -0.430 | 0.678 |
| Group | 3.092 (0.234, 5.950) | 2.137 | 0.072 |
| CSPS | 0.075 (0.009, 0.140) | 2.253 | 0.053 |
| Group x CSPS | -0.082 (-0.160, -0.003) | -2.055 | 0.087 |

*Significant results pFDR <0.05; linear regression with 5,000 permutations, model covariates included gestational age at birth, sex and index of multiple deprivation. Abbreviations: CSPS, cognitively stimulating parenting scale; FDR, false discovery rate; GA, gestational age; IMD, index of multiple deprivation; PC, principal component.

**Supplementary Table S3.** Moderating effect of cognitively stimulating parenting on the association between neonatal SCNs and the “neurodevelopmental difficulties” component (PC1), separately for CHD and controls.

| **SCN** | **CHD (n = 44)** | | | **Controls (n = 117)** | | |
| --- | --- | --- | --- | --- | --- | --- |
|  | **B (95% CI)** | **t-score** | **pFDR** | **B (95% CI)** | **t-score** | **pFDR** |
| (1) CSF around anterior temporal lobe | <0.001  (-0.003, 0.004) | 0.183 | 0.992 | 0.001  (-0.001, 0.003) | 1.152 | 0.405 |
| (2) Anterior temporal region | 0.001  (-0.004, 0.006) | 0.363 | 0.992 | -0.001  (-0.004, 0.002) | -0.771 | 0.637 |
| (3) Caudate + lentiform nucleus | 0.004  (-0.003, 0.011) | 1.069 | 0.848 | -0.003  (-0.007, <0.001) | -1.757 | 0.205 |
| (4) Temporal operculum + anterior frontal WM | -0.007  (-0.015, <0.001) | -1.993 | 0.239 | -0.002  (-0.005, <0.001) | -2.124 | 0.164 |
| (5) Posterior lateral ventricles | 0.001  (-0.003, 0.005) | 0.490 | 0.992 | 0.002  (<0.001, 0.004) | 1.870 | 0.186 |
| (6) Anterior frontal region | 0.001  (-0.004, 0.007) | 0.532 | 0.992 | 0.003  (<0.001, 0.005) | 2.097 | 0.164 |
| (7) Superior cerebellum, pons, CSF around hippocampus | -0.003  (-0.011, 0.005) | -0.704 | 0.992 | <0.001  (-0.002, 0.003) | 0.242 | 0.918 |
| (8) Medial cerebellum | -0.002  (-0.008, 0.003) | -0.902 | 0.980 | -0.003  (-0.006, <0.001) | -2.079 | 0.164 |
| (9) Left visual cortex | -0.002  (-0.008, 0.003) | -0.948 | 0.980 | -0.002  (-0.004, <0.001) | -2.314 | 0.164 |
| (10) Cerebellar CSF | 0.003  (-0.002, 0.008) | 1.178 | 0.830 | -0.004  (-0.007, -0.001) | -2.067 | 0.080 |
| (11) Cerebellum + brainstem | 0.003  (-0.005, 0.011) | 0.679 | 0.992 | <0.001  (-0.003, 0.004) | 0.272 | 0.918 |
| (12) Frontal WM region | <0.001  (-0.004, 0.004) | 0.110 | 0.992 | <0.001  (-0.002, 0.003) | 0.298 | 0.918 |
| (13) Medial motor cortex | 0.004  (-0.001, 0.008) | 1.784 | 0.347 | 0.001  (-0.001, 0.002) | 0.792 | 0.631 |
| (14) Occipito-cerebellar CSF + splenium | 0.001  (-0.004, 0.006) | 0.408 | 0.992 | <0.001  (-0.002, 0.002) | -0.013 | 0.990 |
| (15) Anterior cingulate | -0.002  (-0.006, 0.002) | -1.126 | 0.830 | 0.001  (-0.001, 0.003) | 1.143 | 0.405 |
| (16) Medial occipital region | -0.001  (-0.003, 0.002) | -0.497 | 0.992 | -0.002  (-0.004, <0.001) | -2.072 | 0.164 |
| (17) Somatosensory cortex | -0.002  (-0.006, 0.002) | -1.012 | 0.892 | -0.001  (-0.003, 0.002) | -0.597 | 0.709 |
| (18) Parietal-occipital region | 0.002  (-0.002, 0.006) | 1.191 | 0.793 | <0.001  (-0.002, 0.002) | 0.110 | 0.934 |
| (19) Anterior lateral ventricles | <0.001  (-0.005, 0.004) | -0.214 | 0.992 | 0.003  (<0.001, 0.006) | 2.040 | 0.169 |
| (20) Posterior thalamus + hippocampus | 0.003  (-0.005, 0.012) | 0.764 | 0.992 | -0.004  (-0.007, -0.002) | -2.096 | 0.080 |
| (21) CSF around insula | -0.002  (-0.006, 0.002) | -0.898 | 0.991 | <0.001  (-0.001, 0.002) | 0.166 | 0.923 |
| (22) Inferior frontal region | -0.004  (-0.012, 0.003) | -1.121 | 0.830 | -0.002  (-0.005, <0.001) | -1.965 | 0.169 |
| (23) Motor cortex | 0.002  (-0.005, 0.008) | 0.493 | 0.992 | 0.002  (-0.001, 0.004) | 1.190 | 0.405 |
| (24) Frontal midline (left) | -0.003  (-0.010, 0.004) | -0.935 | 0.980 | 0.002  (<0.001, 0.004) | 1.817 | 0.200 |
| (25) Inferior frontal gyrus | -0.002  (-0.006, 0.003) | -0.720 | 0.992 | 0.001  (-0.001, 0.004) | 1.051 | 0.440 |
| (26) Insula | -0.004  (-0.011, 0.002) | -1.355 | 0.683 | 0.001  (-0.002, 0.003) | 0.510 | 0.787 |
| (27) Orbitofrontal region (right) | 0.002  (-0.004, 0.009) | 0.761 | 0.992 | <0.001  (-0.002, 0.003) | 0.215 | 0.918 |
| (28) Anterior infratentorial + orbitofrontal CSF | <0.001  (-0.006, 0.006) | 0.040 | 0.980 | -0.003  (-0.006, <0.001) | -1.915 | 0.169 |
| (29) Superior frontal CSF | <0.001  (-0.005, 0.005) | -0.026 | 0.980 | 0.002  (<0.001, 0.004) | 2.194 | 0.164 |
| (30) Anterior thalamus + cingulum | 0.007  (-0.002, 0.016) | 1.620 | 0.455 | -0.006  (-0.009, -0.002) | -2.074 | 0.080 |
| (31) Infratentorial CSF | 0.002  (-0.003, 0.008) | 0.832 | 0.992 | -0.002  (-0.005, 0.001) | -1.384 | 0.342 |
| (32) Fusiform gyrus | -0.001  (-0.009, 0.006) | -0.352 | 0.992 | -0.002  (-0.004, 0.001) | -1.515 | 0.320 |
| (33) Inferior temporal gyrus – posterior (left) | -0.003  (-0.009, 0.003) | -0.978 | 0.933 | 0.002  (-0.001, 0.005) | 1.393 | 0.342 |
| (34) Middle frontal gyrus | -0.005  (-0.013, 0.003) | -1.165 | 0.830 | -0.001  (-0.003, 0.001) | -1.326 | 0.370 |
| (35) Inferior temporal gyrus – anterior | 0.002  (-0.003, 0.008) | 0.842 | 0.992 | 0.003  (<0.001, 0.005) | 2.197 | 0.164 |
| (36) Posterior parietal region | 0.002  (-0.002, 0.006) | 1.156 | 0.830 | 0.001  (<0.001, 0.003) | 1.465 | 0.327 |
| (37) Superior temporal gyrus | 0.001  (-0.004, 0.007) | 0.522 | 0.992 | 0.001  (-0.001, 0.004) | 1.244 | 0.405 |
| (38) Precuneus | -0.002  (-0.004, 0.001) | -1.071 | 0.848 | <0.001  (-0.002, 0.003) | 0.183 | 0.919 |
| (39) Inferior temporal gyrus – posterior (right) | -0.003  (-0.009, 0.002) | -1.147 | 0.830 | 0.001  (-0.002, 0.003) | 0.644 | 0.709 |
| (40) Periventricular crossroads | -0.004  (-0.008, 0.001) | -1.709 | 0.366 | <0.001  (-0.002, 0.003) | 0.250 | 0.918 |

Abbreviations: FDR, false discovery rate; PC, principal component; SCN, structural covariance network.

**Supplementary Table S4.** Moderating effect of cognitively stimulating parenting on the association between neonatal SCNs and the “emotional dysregulation” component (PC2), separately for CHD and controls.

| **SCN** | **CHD (n = 44)** | | | **Controls (n = 117)** | | |
| --- | --- | --- | --- | --- | --- | --- |
|  | **B (95% CI)** | **t-score** | **pFDR** | **B (95% CI)** | **t-score** | **pFDR** |
| (1) CSF around anterior temporal lobe | 0.001  (-0.003, 0.005) | 0.570 | 0.757 | 0.001  (-0.001, 0.003) | 0.587 | 0.672 |
| (2) Anterior temporal region | -0.002  (-0.007, 0.003) | -0.652 | 0.748 | <0.001  (-0.003, 0.003) | -0.096 | 0.946 |
| (3) Caudate + lentiform nucleus | -0.005  (-0.012, 0.002) | -1.422 | 0.748 | -0.001  (-0.004, 0.003) | -0.345 | 0.819 |
| (4) Temporal operculum + anterior frontal WM | -0.002  (-0.009, 0.006) | -0.431 | 0.811 | -0.001  (-0.003, 0.002) | -0.634 | 0.669 |
| (5) Posterior lateral ventricles | 0.001  (-0.002, 0.005) | 0.736 | 0.748 | -0.001  (-0.004, 0.001) | -1.147 | 0.520 |
| (6) Anterior frontal region | 0.001  (-0.005, 0.006) | 0.246 | 0.897 | <0.001  (-0.002, 0.003) | 0.311 | 0.830 |
| (7) Superior cerebellum, pons, CSF around hippocampus | -0.005  (-0.012, 0.003) | -1.316 | 0.748 | 0.001  (-0.001, 0.004) | 1.008 | 0.547 |
| (8) Medial cerebellum | -0.004  (-0.009, 0.001) | -1.729 | 0.748 | <0.001  (-0.003, 0.003) | -0.010 | 0.994 |
| (9) Left visual cortex | -0.003  (-0.008, 0.002) | -1.215 | 0.748 | 0.002  (<0.001, 0.004) | 1.564 | 0.465 |
| (10) Cerebellar CSF | -0.003  (-0.008, 0.002) | -1.306 | 0.748 | 0.002  (-0.002, 0.005) | 0.991 | 0.547 |
| (11) Cerebellum + brainstem | 0.003  (-0.006, 0.011) | 0.634 | 0.752 | -0.002  (-0.005, 0.002) | -0.853 | 0.570 |
| (12) Frontal WM region | -0.002  (-0.006, 0.002) | -0.959 | 0.748 | 0.002  (<0.001, 0.005) | 1.737 | 0.378 |
| (13) Medial motor cortex | -0.004  (-0.009, 0.001) | -1.780 | 0.748 | -0.001  (-0.002, 0.001) | -0.565 | 0.672 |
| (14) Occipito-cerebellar CSF + splenium | 0.001  (-0.004, 0.006) | 0.521 | 0.771 | <0.001  (-0.002, 0.002) | 0.114 | 0.943 |
| (15) Anterior cingulate | 0.001  (-0.003, 0.005) | 0.402 | 0.818 | <0.001  (-0.002, 0.002) | 0.125 | 0.931 |
| (16) Medial occipital region | -0.002  (-0.004, 0.001) | -1.321 | 0.748 | <0.001  (-0.002, 0.003) | 0.327 | 0.824 |
| (17) Somatosensory cortex | -0.001  (-0.005, 0.003) | -0.546 | 0.771 | <0.001  (-0.002, 0.002) | -0.088 | 0.946 |
| (18) Parietal-occipital region | 0.001  (-0.003, 0.005) | 0.715 | 0.748 | 0.001  (-0.002, 0.003) | 0.652 | 0.665 |
| (19) Anterior lateral ventricles | <0.001  (-0.004, 0.004) | -0.172 | 0.931 | -0.002  (-0.006, 0.001) | -1.358 | 0.479 |
| (20) Posterior thalamus + hippocampus | -0.003  (-0.012, 0.006) | -0.762 | 0.748 | 0.002  (-0.001, 0.006) | 1.452 | 0.465 |
| (21) CSF around insula | 0.001  (-0.003, 0.005) | 0.451 | 0.793 | -0.001  (-0.002, 0.001) | -0.595 | 0.671 |
| (22) Inferior frontal region | <0.001  (-0.008, 0.008) | 0.009 | 0.998 | 0.001  (-0.001, 0.004) | 0.842 | 0.575 |
| (23) Motor cortex | -0.006  (-0.012, <0.001) | -2.016 | 0.748 | -0.001  (-0.004, 0.002) | -0.974 | 0.547 |
| (24) Frontal midline (left) | <0.001  (-0.007, 0.006) | -0.120 | 0.950 | <0.001  (-0.002, 0.003) | 0.255 | 0.852 |
| (25) Inferior frontal gyrus | <0.001  (-0.005, 0.004) | -0.143 | 0.942 | 0.002  (-0.001, 0.004) | 1.363 | 0.478 |
| (26) Insula | 0.002  (-0.004, 0.009) | 0.697 | 0.748 | 0.002  (-0.001, 0.005) | 1.256 | 0.520 |
| (27) Orbitofrontal region (right) | <0.001  (-0.007, 0.006) | -0.110 | 0.953 | <0.001  (-0.003, 0.002) | -0.271 | 0.852 |
| (28) Anterior infratentorial + orbitofrontal CSF | <0.001  (-0.006, 0.006) | 0.014 | 0.998 | 0.002  (-0.002, 0.005) | 0.956 | 0.554 |
| (29) Superior frontal CSF | 0.001  (-0.004, 0.006) | 0.316 | 0.871 | <0.001  (-0.002, 0.002) | -0.043 | 0.975 |
| (30) Anterior thalamus + cingulum | 0.002  (-0.007, 0.011) | 0.409 | 0.818 | <0.001  (-0.005, 0.004) | -0.190 | 0.898 |
| (31) Infratentorial CSF | -0.002  (-0.008, 0.003) | -0.824 | 0.748 | <0.001  (-0.003, 0.003) | 0.126 | 0.929 |
| (32) Fusiform gyrus | 0.003  (-0.005, 0.011) | 0.780 | 0.748 | 0.001  (-0.001, 0.004) | 1.139 | 0.520 |
| (33) Inferior temporal gyrus – posterior (left) | 0.001  (-0.005, 0.007) | 0.382 | 0.826 | -0.001  (-0.004, 0.002) | -0.881 | 0.554 |
| (34) Middle frontal gyrus | -0.004  (-0.012, 0.004) | -0.955 | 0.748 | 0.001  (-0.001, 0.003) | 0.800 | 0.595 |
| (35) Inferior temporal gyrus – anterior | 0.002  (-0.004, 0.007) | 0.620 | 0.754 | -0.001  (-0.004, 0.001) | -0.900 | 0.560 |
| (36) Posterior parietal region | -0.001  (-0.005, 0.003) | -0.610 | 0.754 | -0.001  (-0.003, 0.001) | -1.016 | 0.547 |
| (37) Superior temporal gyrus | -0.004  (-0.009, 0.001) | -1.819 | 0.748 | -0.001  (-0.003, 0.002) | -0.615 | 0.671 |
| (38) Precuneus | 0.002  (-0.001, 0.005) | 1.387 | 0.748 | 0.001  (-0.002, 0.003) | 0.431 | 0.775 |
| (39) Inferior temporal gyrus – posterior (right) | 0.003  (-0.002, 0.009) | 1.241 | 0.748 | -0.001  (-0.003, 0.002) | -0.693 | 0.648 |
| (40) Periventricular crossroads | <0.001  (-0.005, 0.004) | -0.177 | 0.931 | <0.001  (-0.002, 0.003) | 0.285 | 0.839 |

Abbreviations: FDR, false discovery rate; PC, principal component; SCN, structural covariance network.

**Supplementary Table S5.** Moderating effect of cognitively stimulating parenting on the association between neonatal SCNs and the “social function” component (PC3), separately for CHD and controls.

| **SCN** | **CHD (n = 44)** | | | **Controls (n = 117)** | | |
| --- | --- | --- | --- | --- | --- | --- |
|  | **B (95% CI)** | **t-score** | **pFDR** | **B (95% CI)** | **t-score** | **pFDR** |
| (1) CSF around anterior temporal lobe | <0.001  (-0.003, 0.003) | -0.043 | 0.982 | -0.001  (-0.003, 0.002) | -0.559 | 0.802 |
| (2) Anterior temporal region | 0.002  (-0.002, 0.006) | 0.802 | 0.620 | -0.003  (-0.006, <0.001) | -1.707 | 0.347 |
| (3) Caudate + lentiform nucleus | -0.003  (-0.008, 0.003) | -0.944 | 0.603 | 0.001  (-0.003, 0.005) | 0.685 | 0.745 |
| (4) Temporal operculum + anterior frontal WM | -0.006  (-0.012, <0.001) | -2.131 | 0.287 | 0.001  (-0.002, 0.003) | 0.518 | 0.814 |
| (5) Posterior lateral ventricles | 0.001  (-0.002, 0.004) | 0.537 | 0.746 | -0.001  (-0.003, 0.002) | -0.543 | 0.806 |
| (6) Anterior frontal region | 0.002  (-0.002, 0.006) | 1.056 | 0.558 | 0.001  (-0.002, 0.004) | 0.563 | 0.806 |
| (7) Superior cerebellum, pons, CSF around hippocampus | -0.006  (-0.012, -0.001) | -2.207 | 0.205 | <0.001  (-0.003, 0.003) | -0.025 | 0.990 |
| (8) Medial cerebellum | -0.002  (-0.006, 0.002) | -0.916 | 0.603 | 0.002  (-0.001, 0.005) | 1.291 | 0.510 |
| (9) Left visual cortex | 0.002  (-0.002, 0.007) | 1.172 | 0.496 | 0.001  (-0.001, 0.003) | 0.923 | 0.660 |
| (10) Cerebellar CSF | 0.002  (-0.002, 0.007) | 1.163 | 0.507 | <0.001  (-0.003, 0.004) | 0.036 | 0.986 |
| (11) Cerebellum + brainstem | -0.001  (-0.007, 0.006) | -0.251 | 0.887 | -0.003  (-0.007, 0.001) | -1.535 | 0.390 |
| (12) Frontal WM region | 0.003  (<0.001, 0.005) | 1.792 | 0.312 | -0.001  (-0.004, 0.001) | -1.025 | 0.614 |
| (13) Medial motor cortex | -0.001  (-0.005, 0.003) | -0.489 | 0.772 | 0.001  (-0.001, 0.003) | 1.364 | 0.458 |
| (14) Occipito-cerebellar CSF + splenium | -0.002  (-0.006, 0.003) | -0.781 | 0.623 | -0.003  (-0.005, <0.001) | -2.136 | 0.250 |
| (15) Anterior cingulate | <0.001  (-0.003, 0.003) | -0.251 | 0.882 | 0.002  (<0.001, 0.004) | 1.798 | 0.346 |
| (16) Medial occipital region | <0.001  (-0.002, 0.002) | 0.295 | 0.858 | -0.002  (-0.004, 0.001) | -1.254 | 0.520 |
| (17) Somatosensory cortex | <0.001  (-0.003, 0.003) | 0.289 | 0.863 | 0.001  (-0.001, 0.004) | 0.898 | 0.660 |
| (18) Parietal-occipital region | 0.003  (<0.001, 0.006) | 1.773 | 0.312 | -0.001  (-0.004, 0.001) | -1.101 | 0.597 |
| (19) Anterior lateral ventricles | 0.002  (-0.001, 0.005) | 1.027 | 0.569 | <0.001  (-0.003, 0.004) | 0.226 | 0.919 |
| (20) Posterior thalamus + hippocampus | -0.005  (-0.012, 0.002) | -1.347 | 0.420 | -0.003  (-0.006, <0.001) | -1.767 | 0.347 |
| (21) CSF around insula | -0.002  (-0.006, 0.001) | -1.422 | 0.365 | -0.001  (-0.002, 0.001) | -0.722 | 0.744 |
| (22) Inferior frontal region | 0.002  (-0.004, 0.008) | 0.529 | 0.752 | 0.001  (-0.002, 0.004) | 0.751 | 0.737 |
| (23) Motor cortex | -0.002  (-0.007, 0.003) | -0.849 | 0.620 | <0.001  (-0.003, 0.003) | 0.023 | 0.991 |
| (24) Frontal midline (left) | -0.003  (-0.008, 0.002) | -1.124 | 0.521 | 0.001  (-0.001, 0.004) | 1.135 | 0.574 |
| (25) Inferior frontal gyrus | -0.003  (-0.006, <0.001) | -1.745 | 0.312 | <0.001  (-0.003, 0.002) | -0.231 | 0.919 |
| (26) Insula | 0.001  (-0.004, 0.006) | 0.450 | 0.790 | 0.002  (-0.001, 0.005) | 1.586 | 0.370 |
| (27) Orbitofrontal region (right) | 0.002  (-0.003, 0.007) | 0.883 | 0.620 | -0.002  (-0.005, 0.001) | -1.117 | 0.574 |
| (28) Anterior infratentorial + orbitofrontal CSF | 0.002  (-0.003, 0.007) | 0.726 | 0.634 | <0.001  (-0.003, 0.004) | 0.190 | 0.925 |
| (29) Superior frontal CSF | 0.001  (-0.003, 0.004) | 0.322 | 0.852 | 0.001  (-0.001, 0.003) | 1.052 | 0.608 |
| (30) Anterior thalamus + cingulum | -0.003  (-0.010, 0.005) | -0.745 | 0.630 | -0.001  (-0.006, 0.003) | -0.488 | 0.814 |
| (31) Infratentorial CSF | <0.001  (-0.005, 0.005) | 0.041 | 0.982 | 0.001  (-0.002, 0.005) | 0.919 | 0.660 |
| (32) Fusiform gyrus | -0.001  (-0.007, 0.005) | -0.367 | 0.826 | 0.001  (-0.002, 0.004) | 0.682 | 0.745 |
| (33) Inferior temporal gyrus – posterior (left) | 0.005  (<0.001, 0.009) | 2.212 | 0.231 | 0.002  (-0.001, 0.006) | 1.493 | 0.412 |
| (34) Middle frontal gyrus | -0.005  (-0.011, 0.002) | -1.464 | 0.365 | -0.001  (-0.003, 0.001) | -0.565 | 0.802 |
| (35) Inferior temporal gyrus – anterior | 0.005  (0.001, 0.008) | 2.466 | 0.158 | <0.001  (-0.003, 0.002) | -0.262 | 0.909 |
| (36) Posterior parietal region | -0.002  (-0.005, 0.002) | -0.989 | 0.581 | 0.001  (-0.001, 0.003) | 0.929 | 0.650 |
| (37) Superior temporal gyrus | 0.003  (<0.001, 0.007) | 1.837 | 0.312 | -0.001  (-0.004, 0.001) | -1.202 | 0.550 |
| (38) Precuneus | <0.001  (-0.002, 0.002) | -0.168 | 0.929 | <0.001  (-0.002, 0.003) | 0.277 | 0.909 |
| (39) Inferior temporal gyrus – posterior (right) | 0.003  (-0.001, 0.008) | 1.554 | 0.353 | 0.001  (-0.001, 0.004) | 1.049 | 0.608 |
| (40) Periventricular crossroads | -0.001  (-0.005, 0.003) | -0.517 | 0.760 | 0.001  (-0.001, 0.004) | 1.136 | 0.582 |

Abbreviations: FDR, false discovery rate; PC, principal component; SCN, structural covariance network.

**Supplementary Table S6.** Moderating effect of cognitively stimulating parenting on the association between neonatal SCNs and the “empathy” component (PC4), separately for CHD and controls.

| **SCN** | **CHD (n = 44)** | | | **Controls (n = 117)** | | |
| --- | --- | --- | --- | --- | --- | --- |
|  | **B (95% CI)** | **t-score** | **pFDR** | **B (95% CI)** | **t-score** | **pFDR** |
| (1) CSF around anterior temporal lobe | <-0.001  (-0.005, 0.004) | -0.039 | 0.996 | 0.002  (<-0.001, 0.004) | 1.871 | 0.542 |
| (2) Anterior temporal region | -0.002  (-0.008, 0.004) | -0.661 | 0.791 | <-0.001  (-0.003, 0.003) | -0.183 | 0.927 |
| (3) Caudate + lentiform nucleus | -0.007  (-0.015, 0.002) | -1.507 | 0.704 | 0.002  (-0.001, 0.006) | 1.146 | 0.731 |
| (4) Temporal operculum + anterior frontal WM | -0.006  (-0.015, 0.004) | -1.226 | 0.704 | -0.002  (-0.004, <0.001) | -1.606 | 0.542 |
| (5) Posterior lateral ventricles | 0.001  (-0.003, 0.006) | 0.543 | 0.873 | -0.001  (-0.003, 0.001) | -0.98 | 0.814 |
| (6) Anterior frontal region | 0.001  (-0.005, 0.008) | 0.378 | 0.913 | <-0.001  (-0.003, 0.002) | -0.078 | 0.937 |
| (7) Superior cerebellum, pons, CSF around hippocampus | -0.002  (-0.012, 0.007) | -0.513 | 0.880 | 0.002  (<-0.001, 0.005) | 1.862 | 0.542 |
| (8) Medial cerebellum | -0.002  (-0.009, 0.004) | -0.711 | 0.773 | -0.001  (-0.004, 0.002) | -0.556 | 0.826 |
| (9) Left visual cortex | 0.002  (-0.004, 0.009) | 0.687 | 0.773 | -0.001  (-0.003, 0.001) | -0.794 | 0.814 |
| (10) Cerebellar CSF | 0.008  (0.002, 0.014) | 2.527 | 0.563 | <0.001  (-0.003, 0.003) | 0.075 | 0.937 |
| (11) Cerebellum + brainstem | -0.008  (-0.018, 0.002) | -1.563 | 0.704 | 0.003  (<0.001, 0.007) | 1.983 | 0.542 |
| (12) Frontal WM region | 0.001  (-0.003, 0.006) | 0.53 | 0.868 | 0.002  (-0.001, 0.004) | 1.441 | 0.542 |
| (13) Medial motor cortex | -0.004  (-0.009, 0.002) | -1.209 | 0.704 | <-0.001  (-0.002, 0.001) | -0.547 | 0.826 |
| (14) Occipito-cerebellar CSF + splenium | -0.002  (-0.009, 0.004) | -0.719 | 0.773 | -0.002  (-0.004, 0.001) | -1.501 | 0.542 |
| (15) Anterior cingulate | <0.001  (-0.005, 0.005) | 0.085 | 0.996 | -0.001  (-0.004, 0.001) | -1.372 | 0.581 |
| (16) Medial occipital region | -0.001  (-0.004, 0.002) | -0.53 | 0.868 | -0.001  (-0.003, 0.002) | -0.567 | 0.826 |
| (17) Somatosensory cortex | 0.002  (-0.003, 0.007) | 0.821 | 0.740 | <0.001  (-0.002, 0.002) | 0.212 | 0.927 |
| (18) Parietal-occipital region | <-0.001  (-0.005, 0.005) | -0.045 | 0.996 | 0.001  (-0.001, 0.003) | 0.881 | 0.814 |
| (19) Anterior lateral ventricles | 0.002  (-0.003, 0.007) | 0.728 | 0.773 | -0.002  (-0.005, 0.001) | -1.206 | 0.688 |
| (20) Posterior thalamus + hippocampus | -0.002  (-0.013, 0.010) | -0.297 | 0.939 | 0.003  (<0.001, 0.006) | 2.080 | 0.542 |
| (21) CSF around insula | -0.001  (-0.007, 0.004) | -0.496 | 0.879 | <-0.001  (-0.002, 0.001) | -0.287 | 0.912 |
| (22) Inferior frontal region | -0.001  (-0.011, 0.008) | -0.234 | 0.966 | <-0.001  (-0.003, 0.002) | -0.084 | 0.937 |
| (23) Motor cortex | 0.001  (-0.007, 0.009) | 0.218 | 0.981 | <0.001  (-0.002, 0.003) | 0.306 | 0.912 |
| (24) Frontal midline (left) | 0.009  (<0.001, 0.017) | 2.128 | 0.569 | 0.002  (-<0.001, 0.004) | 1.548 | 0.542 |
| (25) Inferior frontal gyrus | -0.002  (-0.008, 0.003) | -0.905 | 0.718 | -0.001  (-0.003, 0.002) | -0.732 | 0.826 |
| (26) Insula | -0.004  (-0.012, 0.004) | -1.051 | 0.704 | -0.001  (-0.004, 0.001) | -1.065 | 0.805 |
| (27) Orbitofrontal region (right) | -0.005  (-0.013, 0.002) | -1.432 | 0.704 | <0.001  (-0.002, 0.003) | 0.243 | 0.924 |
| (28) Anterior infratentorial + orbitofrontal CSF | 0.005  (-0.002, 0.013) | 1.468 | 0.704 | 0.001  (-0.002, 0.004) | 0.45 | 0.862 |
| (29) Superior frontal CSF | 0.004  (-0.002, 0.010) | 1.302 | 0.704 | 0.002  (-0.001, 0.004) | 1.447 | 0.542 |
| (30) Anterior thalamus + cingulum | 0.002  (-0.009, 0.012) | 0.327 | 0.933 | -0.001  (-0.005, 0.003) | -0.398 | 0.888 |
| (31) Infratentorial CSF | <0.001  (-0.007, 0.007) | 0.047 | 0.996 | -0.001  (-0.004, 0.002) | -0.857 | 0.814 |
| (32) Fusiform gyrus | -0.005  (-0.015, 0.004) | -1.179 | 0.704 | -0.001  (-0.003, 0.002) | -0.56 | 0.826 |
| (33) Inferior temporal gyrus - posterior (left) | 0.007  (<-0.001, 0.014) | 1.907 | 0.569 | -0.001  (-0.004, 0.002) | -0.66 | 0.826 |
| (34) Middle frontal gyrus | -0.004  (-0.014, 0.007) | -0.73 | 0.773 | <0.001  (-0.001, 0.002) | 0.45 | 0.862 |
| (35) Inferior temporal gyrus - anterior | 0.011  (0.005, 0.016) | 3.72 | **0.040*** | 0.001  (-0.001, 0.003) | 0.797 | 0.814 |
| (36) Posterior parietal region | -0.003  (-0.008, 0.002) | -1.404 | 0.704 | <0.001  (-0.001, 0.002) | 0.348 | 0.899 |
| (37) Superior temporal gyrus | 0.007  (0.001, 0.013) | 3.418 | **0.048*** | 0.003  (0.001, 0.005) | 3.390 | **0.047*** |
| (38) Precuneus | -0.001  (-0.005, 0.003) | -0.548 | 0.868 | 0.001  (-0.001, 0.003) | 0.919 | 0.814 |
| (39) Inferior temporal gyrus - posterior (right) | 0.007  (<0.001, 0.014) | 2.131 | 0.569 | 0.002  (-0.001, 0.004) | 1.494 | 0.542 |
| (40) Periventricular crossroads | -0.002  (-0.007, 0.004) | -0.648 | 0.795 | -0.001  (-0.003, 0.002) | -0.628 | 0.826 |

*Significant results pFDR <0.05; linear regression with 5,000 permutations, model covariates included gestational age at birth, postmenstrual age at scan, sex and index of multiple deprivation. Abbreviations: FDR, false discovery rate; PC, principal component; SCN, structural covariance network.
